# Evolving prion-like tau conformers differentially alter postsynaptic proteins in neurons inoculated with distinct isolates of Alzheimer’s disease tau

**DOI:** 10.1186/s13578-023-01133-0

**Published:** 2023-09-18

**Authors:** Lenka Hromadkova, Chae Kim, Tracy Haldiman, Lihua Peng, Xiongwei Zhu, Mark Cohen, Rohan de Silva, Jiri G. Safar

**Affiliations:** 1 Departments of Pathology, Case Western Reserve University School of Medicine, 2085 Adelbert Rd, Cleveland, OH 44106 USA; 2 Departments of Neurology, Case Western Reserve University School of Medicine, Cleveland, OH 44106 USA; 3 Departments of Neuroscience, Case Western Reserve University School of Medicine, Cleveland, OH 44106 USA; 4 National Prion Disease Pathology Surveillance Center, Case Western Reserve University School of Medicine, Cleveland, OH 44106 USA; 5 Reta Lila Weston Institute, UCL Queen Square Institute of Neurology, London, WC1N 1PJ UK

**Keywords:** Alzheimer’s disease, Conformational diversity, Postsynaptic scaffolding proteins, Propagation of tau aggregation, Synapses loss, Tau misfolding, Tau protein

## Abstract

**Objectives:**

Although accumulation of misfolded tau species has been shown to predict cognitive decline in patients with Alzheimer’s disease (AD) and other tauopathies but with the remarkable diversity of clinical manifestations, neuropathology profiles, and time courses of disease progression remaining unexplained by current genetic data. We considered the diversity of misfolded tau conformers present in individual AD cases as an underlying driver of the phenotypic variations of AD and progressive loss of synapses.

**Methods:**

To model the mechanism of tau propagation and synaptic toxicity of distinct tau conformers, we inoculated wild-type primary mouse neurons with structurally characterized Sarkosyl-insoluble tau isolates from the frontal cortex of six AD cases and monitored the impact for fourteen days. We analyzed the accumulation rate, tau isoform ratio, and conformational characteristics of de novo-induced tau aggregates with conformationally sensitive immunoassays, and the dynamics of synapse formation, maintenance, and their loss using a panel of pre-and post-synaptic markers.

**Results:**

At the same concentrations of tau, the different AD tau isolates induced accumulation of misfolded predominantly 4-repeat tau aggregates at different rates in mature neurons, and demonstrated distinct conformational characteristics corresponding to the original AD brain tau. The time-course of the formation of misfolded tau aggregates and colocalization correlated with significant loss of synapses in tau-inoculated cell cultures and the reduction of synaptic connections implicated the disruption of postsynaptic compartment as an early event.

**Conclusions:**

The data obtained with mature neurons expressing physiological levels and adult isoforms of tau protein demonstrate markedly different time courses of endogenous tau misfolding and differential patterns of post-synaptic alterations. These and previous biophysical data argue for an ensemble of various misfolded tau aggregates in individual AD brains and template propagation of their homologous conformations in neurons with different rates and primarily postsynaptic interactors. Modeling tau aggregation in mature differentiated neurons provides a platform for investigating divergent molecular mechanisms of tau strain propagation and for identifying common structural features of misfolded tau and critical interactors for new therapeutic targets and approaches in AD.

**Supplementary Information:**

The online version contains supplementary material available at 10.1186/s13578-023-01133-0.

## Background

Alzheimer’s disease (AD) is associated with a high degree of heterogeneity in cognitive decline and symptoms’ severity across individual patients [1–3], but the mechanisms leading to distinct clinical phenotypes is not fully understood. Tau protein has been investigated as a candidate responsible for this heterogeneity as tau aggregation is one of the main pathological features of AD [4] and the severity of AD symptoms correlates with tau propagation across the brain tissue [5]. In addition to the link between cognitive decline and tau propagation, increasing dysfunction and synaptic loss are also significantly linked to AD progression [6, 7]. In AD brains, the phosphorylation state and misfolding of tau correlates with reduced levels of both pre- and post-synaptic scaffolding proteins and mis-sorting of pathological tau species leads to gradual loss of synapses [8–11]. The precise molecular mechanisms of the interplay between synaptic proteins and aggregated tau leading to synaptic loss is poorly understood but these fundamental observations indicate that both propagation of tau misfolding and synaptic disruption are intrinsically connected [12–14].

The underlying mechanism of propagation of misfolded tau conformers from a relatively small hub of cells to anatomically connected areas, and the resulting diverse effects implicate differences in structural organization of pathogenic aggregates and prion-like process [15–18]. The recent advances in high-resolution structural studies of tau filaments by cryogenic electron microscopy (cryo-EM) confirmed their conformational diversity in different neurodegenerative disorders with tau pathology [17, 19–22]. Although cryo-EM studies have identified a uniform set of two conformations of insoluble tau fibrils in AD that are clearly distinct from tau fibrillar structures which are present in other tauopathies [23, 24], more recent studies in vitro suggested remarkable conformational heterogeneity of tau aggregates [25]. Recent direct biophysical and mass spectrometry data indicate extensive conformational and post-translational diversity of misfolded tau accumulating in different AD brains [10, 17, 21, 22, 26]. These findings revealed that evolving conformer populations (ensembles) are driving in a prion-like manner the different phenotypes in individual AD, and implicate the prion-like propagation and evolution of different biologically active conformers of tau (strains) across the AD cases [17, 18]. Moreover, different tau conformers displayed distinct seeding potency in in vitro (RT QuIC) and in biosensor cell assay [17]. These observations mirrored our earlier data on tau conformers in Frontotemporal lobar degeneration (FTLD)-MAPT-P301L patients with different clinical phenotypes and in TgTau(P301L) mouse model [27, 28].

Tau biosensor assays and overexpression of tau in neuronal cultures have substantially expanded our knowledge about tau seeding activity and conformational diversity of misfolded tau protein [17, 26, 27, 29–36]. However, these seeding assays do not allow the investigation of molecular mechanisms of late synaptic effects. Substitution of proline in mutant tau (P301S/L) has by itself pro-aggregation effect facilitating assembly into fibrils but introduces species barrier effect for human non-mutant tau [37–40]. Additionally, most of the cell biosensors are exposed to tau aggregates via cationic lipid-based transfection reagents such as lipofectamine to maximize nonselective uptake of tau for short-term inoculation [32, 41], investigation of critical steps in cell entry, propagation, and synaptic toxicity of pathogenic tau proteins calls for an alternative model.

**Fig. 1:**
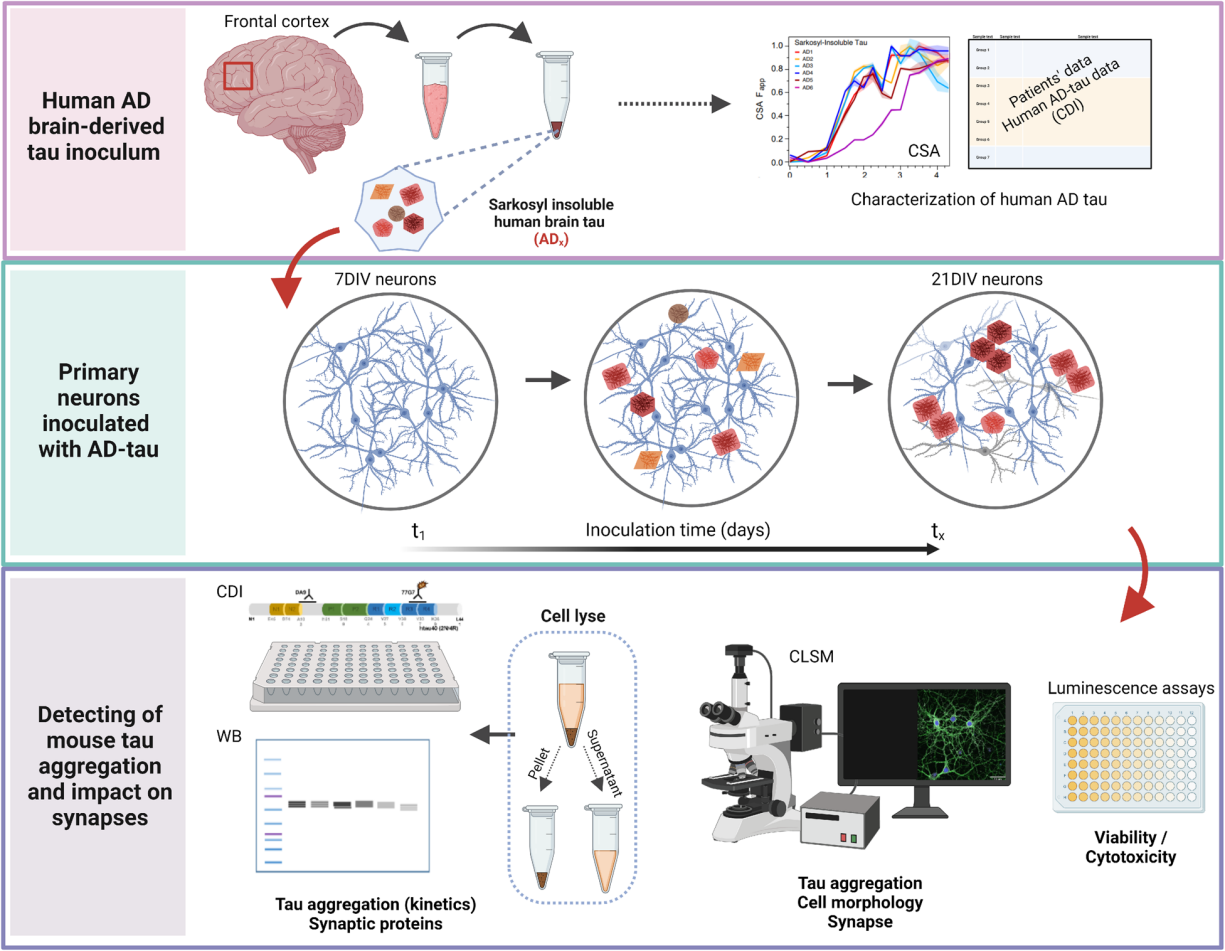
The scheme of the experimental workflow. Human AD brain-derived tau inocula were obtained from AD patients’ frontal cortex, tissue was homogenized, and tau was enriched from sarkosyl-insoluble fraction by sodium phosphotungstate. Tau concentrations and conformational properties in samples were evaluated by conformation-dependent immunoassay (CDI) and conformational stability assay (CSA). Mouse primary neurons were inoculated with human AD-tau samples of the same tau concentration at 7DIV for the maximum inoculation time point at 21DIV to investigate the template propagation of tau misfolding and aggregation. The viability and cytotoxicity were performed to assess tau concentration working range. By confocal microscopy, we investigated mouse tau aggregation after 14 days of inoculation, cell morphology, and the effects on synapse levels. Inoculated neuronal cultures were also lysed, and the concentration, aggregation rate, and conformational properties of sarkosyl-insoluble tau were measured from pellet fractions by CDI and western blots. The supernatants were used in western blot analysis of synaptic markers

Contribution of diverse pools of pathological tau strains with distinct conformational and bioactive properties to the clinicopathological variability of AD is vigorously debated [17, 21, 22, 26]. We applied the prion strain concept to fully differentiated neuronal cultures that were exposed to structurally distinct AD brain-derived tau strains and analyzed the effects with a battery of biophysical and imaging tools (Fig. 1). The data presented here show that various misfolded tau conformers found in the individual AD cases can template mature 4-repeat mouse tau expressed in cortical and hippocampal neurons with conformational fidelity. Consequently, evolution of misfolded tau conformers drives the disruption of post-synaptic terminal structures, preceding major pre-synaptic alterations. The data argue that the differential synaptic loss observed with distinct AD tau strains at later stages is initiated early on with the deterioration of post-synaptic structures. These data support the complexity of molecular mechanisms in AD and the need for patient-to-patient therapeutic interventions and drug discoveries targeting entities highly correlated with disease progression.

## Materials and methods

### Ethics statement

All procedures were performed under protocols approved by the Institutional Review Board at Case Western Reserve University and University Hospitals Case Medical Center in Cleveland, OH. In all cases, written informed consent for research was obtained from the patient or legal guardian, and the material used had appropriate ethical approval for use in this project. All patients’ data and samples were coded and handled according to NIH guidelines to protect patients’ identities.

### Patients and clinical evaluations

The AD cases were randomly selected from a group of AD cases diagnosed between 2001 and 2017 at the Brain Health and Memory Center of the Neurological Institute at University Hospitals Case Medical Center, and Department of Pathology at Case Western Reserve University [17, 42–44], and National Prion Disease Pathology Surveillance Center (NPDPSC) [17, 44]. The criteria for inclusion for AD were: (1) unequivocal clinical diagnosis of AD [45]; (2) absent autosomal dominant pattern of dementia; (3) unequivocal classification as AD after detailed neuropathology and immunohistochemistry of tau proteins and amyloid beta using NIA-AA criteria [46, 47]; and (4) absence of concurrent clinical or neuropathological comorbidity; In all cases, the clinical diagnosis of probable spAD and rpAD was confirmed by diagnostic histopathology [45]. The control age-matched non-neurological group consisted of age- and sex-matched patients whose primary cause of death was lymphoma, carcinomatosis, or autoimmune disorder and the neuropathology ruled out prion disease, AD, or other neurodegenerative disorder.

### Sequencing of APOE genes

DNA was extracted from frozen brain tissues in all cases, and genotypic analysis of the APOE gene polymorphism and the PRNP coding region was performed as described [48, 49]. The coding regions of APP, PSEN1 and PSEN2 were analyzed using a TruSeq Custom Amplicon kit generated by DesignStudio (www.Illumina.com) and were reported previously [44]. Screening for APOE alleles and exons 4 and 5 in PSEN1 were carried out by polymerase chain reaction followed by Sanger dideoxy sequencing as described previously [44, 50].

### Brain sampling

Coronal sections of human brain tissues were obtained at autopsy and stored at − 80ºC. Slices of frontal cortex (superior and more posterior middle gyri) weighing 200–350 mg were homogenized to a final 15% (w/v) concentration by three 75 s cycles with Mini-beadbeater 16 Cell Disrupter (Biospec) in PBS/2% Sarkosyl, pH 7.4, and clarified at 500xg for 5 min. at 4 °C. Clear supernatant was then transferred to a new tube and stored for future analysis at − 80 °C.

### Sandwich-formatted CDI for tau strains

The samples of frontal cortex homogenate were diluted to a final 10% (w/v) concentration with PBS containing 2% Sarkosyl, sonicated 3 × 5 s. each at 80% power with Sonicator 4000 (Qsonica), and spun at 15,500 rpm at 4 °C for 30 min in Allegra X-22R tabletop centrifuge (Beckman Coulter) to obtain Sarkosyl-soluble and Sarkosyl-insoluble tau fractions. The supernatant containing Sarkosyl-soluble tau was transferred to a new tube for CDI analysis and stored at − 80 °C. The pellet containing Sarkosyl-insoluble tau was resuspended in PBS, pH 7.4 with protease inhibitors cocktail (0.5 mM PMSF and aprotinin and leupeptin at 5 µg/ml, respectively) then stored at − 80 °C for future analysis.

The CDI was performed as described previously for mammalian prions [49, 51–54], amyloid beta [44], and recently for brain-derived tau [27] with minor modifications. First, we used white Lumitrac 600 High Binding Plates (E&K Scientific) coated with mAb DA9 (epitope 102–139, gift of late Dr. Peter Davies) in 200 mM NaH_2_PO_4_ containing 0.03% (w/v) NaN_3_, pH 7.5. Each resuspended pellet was sonicated 3 × 5 s. each at 80% power with Sonicator 4000 then split into two aliquots: the first one was denatured (D) with final concentration of 4 M Gdn HCl and the second one, native (N), was untreated. Aliquots of 20 µl from each aliquot containing 0.007% (v/v) of Patent Blue V (Sigma) were directly loaded into wells of white strip plates prefilled with 200 µl of either casein/0.05% Tween20 in TBS, pH 7.4 (SurModics) for denatured aliquot or casein/0.05% Tween20 in TBS, pH 7.4/0.4 M Gdn HCl for native aliquot. Finally, the captured tau was detected by a Europium-conjugated [51] anti-tau mAb 77G7 (epitope 316–355 of 2N4R tau; Biolegend) and mAb RD3 (clone 8E6/C11) (epitope 267–316; [55, 56]); the time-resolved fluorescence (TRF) signals of europium was measured by the multi-mode microplate reader PHERAstar Plus (BMG LabTech). The Eu-N1 ITC (Perkin Elmer) labeling was performed as we described previously [51] with a final Eu/IgG molar ratio of 4.4 for 77G7 and 3.8 for RD3, respectively. The recombinant 2N4R (tau441) and 2N3R (tau410) splicing variant of human tau expressed in E. Coli (rPeptide, Watkinsville, GA) without His tag [57] was used as a calibrant after complete denaturation in 4 M Gdn HCl. The initial concentration of reduced recombinant human tau441 and tau410 was calculated from the absorbance at 280 nm and molar extinction coefficient 7450 M^−1^ cm^−1^. The purified recombinant proteins were dissolved in 4 M Gdn HCl and 50% Stabilcoat (SurModics), and stored at − 80 °C. The concentrations of total (3R + 4R) tau were calculated from the CDI signal of denatured samples detected with Eu-77G7 mAb and calibration curve created from serially diluted recombinant tau441. The concentration of 3R tau was measured with Eu-RD3 mAb and tau410 calibration curve. The relative proportion of each form is expressed as % of 3R and 4R isoform over sum of both isoforms (4R + 3R tau). The TRF signal of denatured and native sample aliquots is expressed as a ratio (D/N) and is a measure of exposed epitopes in the native state against the reference of fully unfolded protein.

### Monitoring dissociation and unfolding of tau strains by CSA

The sequential denaturation of human tau was performed as described previously for mammalian prions [51, 54, 58, 59], following modifications described recently for tau [27]. The 5% (w/v) brain homogenate in PBS containing 2% Sarkosyl was split in two aliquots, one was incubated with 50 µg/ml of Proteinase K at 37 °C for 1 h at 600 rpm in an Eppendorf Thermomixer, and the second one, untreated, was mixed with a protease inhibitors cocktail (0.5 mM PMSF and aprotinin and leupeptin at 5 µg/ml, respectively). The sarkosyl-insoluble tau in both aliquots was precipitated with 0.64% of Sodium Phosphotungstate and 5 mM MgCl_2_ after incubation for 1 h at 37 °C in Eppendorf Thermomixer as described for prions and tau [51, 54, 60, 61], and the pellets collected at 15,500 rpm at 20 °C for 30 min in Allegra X-22R tabletop centrifuge were resuspended in PBS, pH 7.4 and stored for analysis at − 80 °C.

Frozen aliquots of samples containing Sarkosyl-insoluble and PTA-precipitated tau were thawed, sonicated 3 × 5 s. each at 80% power with Sonicator 4000, and the concentration was adjusted to a constant 3.5 ug/ml of tau. 15 µl aliquots in 15 tubes were treated with increasing concentrations of 8 M Gdn HCl containing 0.007% (v/v) Patent Blue V in 0.25 M or 0.5 M increments. After 30 min incubation at room temperature, individual samples were rapidly diluted with casein/0.05% Tween20 in TBS, pH 7.4 containing diminishing concentrations of 8 M Gdn HCl, so that the final concentration in all samples was 0.411 M. Each individual aliquot was immediately loaded to dry white Lumitrac 600, High Binding Plates, coated with mAb DA9 previously blocked with casein/0.05% Tween20/6% sorbitol/0.03% sodium azide, and developed in accordance with CDI protocol using europium-labeled mAb 77G7 for detection as described for mammalian prions [49, 51–54, 62, 63]. The raw TRF signal was converted into the apparent F_app_ as follows: F_app_ = (TRF_OBS_ -TRF_N_)/(TRF_U_—TRF_N_) where TRF_OBS_ is the observed TRF value, and TRF_N_ and TRF_U_ are the TRF values for native and unfolded forms, respectively, at the given Gdn HCl concentration [54, 59, 64]. To determine the concentration of Gdn HCl where 50% of tau is unfolded ([Gdn HCl]_1/2_), the data were fitted by least square method with a sigmoidal transition model (Eq. 1):

1 $$F_{app} \, = \,F_{0} + \frac{{\left( {F_{\max } - F_{0} } \right)}}{{1 + e^{{\left\{ {\left( {c_{1/2} - c} \right)/r} \right\}}} }}$$

The apparent F_app_ in the TRF signal is the function of Gdn HCl concentration(c); c_1/2_ is the concentration of Gdn HCl at which 50% of tau strains is dissociated/unfolded and r is the slope constant [27]. We used for deconvolution of averaged CSA profiles the multiple peak Gaussian model to identify common components in the CSA curves and the Gdn HCl concentration at the maximum peak height. Using these peak-derived Gdn HCl values, the CSA Fapp values at a given Gdn HCl concentration were compared in individual AD cases using two tailed ANOVA.

### Direct format of CSA for protease-resistant core of tau strains

For the PK-resistant misfolded aggregates of tau, frozen aliquots of PK-treated, Sarkosyl-insoluble and PTA-precipitated tau were thawed, sonicated 3 × 5 s. each at 80% power with Sonicator 4000, and the concentration was adjusted to constant ~ 250–350 ng/ml of tau. 15 µl aliquots in 15 tubes were treated with increasing concentrations of 8 M Gdn HCl in 0.25 M or 0.5 M increments. After 30 min incubation at room temperature, individual tubes were rapidly diluted with H_2_O containing diminishing concentrations of 8 M Gdn HCl, so that the final concentration in all samples was 0.2 M. Each aliquot was immediately loaded in triplicates to dry white Lumitrac 600, High Binding Plates. Following overnight incubation at 4 °C and blocking with casein/0.05% Tween 20/6% sorbitol, the plates were developed with europium-labeled mAb 77G7. The raw time-resolved fluorescence (TRF) signals obtained with the multi-mode microplate reader PHERAstar Plus were converted into the apparent F_app_ and to obtain the concentration of Gdn HCl where 50% of tau is unfolded ([Gdn HCl]_1/2_), the data were fitted by least square method with a sigmoidal transition model as described for sandwich CSA [27].

To fit and deconvolute the non-sigmoidal denaturation profiles, we used statistical mechanical deconvolution and Gaussian models originally developed for proteins that undergo more than one-step thermal denaturation [65]. The Gaussian model was also used to analyze the fractional change after PK: the CSA obtained after PK treatment were subtracted from F_app_ values obtained before PK (ΔF_app_ = F^0^–F^PK^) and then fitted with a Gaussian model to estimate the proportion and average stability of protease-sensitive tau strains conformers [27].

(Eq. 2):

2 $$\Delta F_{app} \, = \,F_{0} + A^{{\left\{ { - \left( {c - c_{0} } \right)^{2} } \right\}}}$$

In this model, the PK-induced fractional change is ΔF_app_, F_0_ is fractional change at 0 concentration of Gdn HCl, and c_0_ is the Gdn HCl concentration at the maximum height A of the peak [27].

### Statistical analysis

Statistical analysis was performed using SPSS Statistics 27 package (IBM) or KaleidaGraph (Synergy) software. Experimental results were expressed as mean ± SEM, and a post hoc test (Dunnet) was used to calculate the P values. Significance level (α) was set to p < 0.05.

### Immunodepletion of tau from AD5-tau inoculum

Tau protein conformers were immunodepleted from a fraction of the AD5-tau sample by combined magnetic immunosorbent prepared separately by binding of biotin-conjugated Tau5 (30 µg, epitope 210–230 aa, BioLegend) and AT8 (30 µg, epitope pSer202/pThr205, Invitrogen) antibodies to Pierce Streptavidin magnetic beads (Invitrogen). Before immunomagnetic separation, 250 µl of AD5-tau sample (14.4 µg tau based on CDI data) was sonicated 3 × 5 s. each at 80% power with Sonicator 4000 and spun at 14,000 rpm in Allegra X-22R tabletop centrifuge for 30 min at 4 °C, the supernatant was added to Tau5/AT8-biotin/streptavidin-magnetic particles complex overnight at 4 °C and the flow-through fraction was kept as the combined immunodepleted AD5-tau sample.

### Culturing mouse primary neurons

Primary cortical and hippocampal neurons were prepared from E16.5–17.5 of wild-type C57Bl6/Tac mice. Brains were removed from fetal mice; cortices and hippocampi were dissected separately to ice-cold PBS, washed by, and transferred to ice-cold HBSS, and treated with papain (20 U/ml, Worthington Biochem Corp.) at 37 °C for 20 min. After, the dissociated tissue was treated with Deoxyribonuclease I from bovine pancreas (Millipore Sigma) in HBSS with 2% FBS and washed by HBSS. Complete neurobasal medium (Gibco) supplemented with 1 × GlutaMAX (Gibco) and B27 (Gibco) was added, and tissue was gently triturated, cells were transferred through cell strainer (pore size: 70 µm, Corning). Primary hippocampal neurons were seeded on poly-D-lysine (PDL) pre-coated 96 well plates (Nunc MicroWell Manufacturer) at a density of 5 × 10^3^ cells per well for cytotoxicity, viability, and microscopy protocols. Primary cortical neurons were seeded on PDL-coated 12 well plates at a density of 2.7 × 10^5^ per well. Every 5–6 days, 20% of the medium was removed and replenished with the fresh complete neurobasal medium supplemented with GlutaMAX and B27. All protocols were approved by the Institution’s Animal Care and Use Committee of Case Western Reserve University, School of Medicine.

### Treatment

All the inoculates including AD-tau, mouse tau (mTau), and heparin-induced K18-tau fibrils samples were kept at − 80 °C, thawed on ice, and sonicated 3 × 5 s. each at 80% power with Sonicator 4000 before adding to complete neurobasal medium. Primary neuronal cultures on day 7 (7DIV) were treated with AD-tau samples in concentrations 5, 15, and 45 ng of AD-tau #1–6 and mTau samples per well in complete neurobasal medium with GlutaMAX and B27 supplements in 96-well plate format and 180 ng/well in 12 well plate format. At 21DIV, primary hippocampal neurons were fixed or applied for ATP assay. Primary cortical neurons were treated for 1 h, 3 days, 7 days, and 14 days before cell lyse. Cultures with complete neurobasal medium serve as reference controls. Heparin-induced K18 fibrils were added to neurons in PDL pre-coated 96 well plates at 7DIV and 18DIV in 0.1 µM and 1 µM concentrations and cultured for 14 and 3 days, respectively.

### Viability and cytotoxicity assessment

CellTiter-Glo 2.0 Assay (Promega, G9242) is a luminescence assay to evaluate cell viability by quantification of ATP, which indicates the metabolic activity of living cells. Primary neurons were cultured in a 96-well plate (5 × 10^3^ cells/100ul per well) and treated at 7DIV. At 21DIV, 14 days after treatment, two wells were treated with Triton X-100 (Millipore Sigma) in a final 0.2% concentration for 20 min in a cell incubator to cause cell death as a negative control for this assay. The medium was discarded, cells were washed once with PBS, and the assay was performed according to the manufacturer’s instructions with slight modifications. The amount of CellTiter-Glo^®^ 2.0 Reagent equal to PBS volume was added, and cells were lysed for 2 min on an orbital shaker and 10 min incubated at room temperature. Half of the wells’ volumes were transferred into light grey half-area Alpha plate-96 (Perkin Elmer) to measure the luminescence signal by PHERAstar. ATP standard calibration curve was generated with every plate by using ATP disodium salt hydrate (Millipore Sigma) as an inter-plate control.

LDH-Glo Cytotoxicity Assay (J2380, Promega) measures the amount of LDH released to media by a bioluminescent method as an indication of plasma membrane disruption. The media from all wells were collected at 7DIV before treatment and at 21DIV after 14 days of treatment and stored in LDH storage buffer (200 mM Tris–HCl pH 7.4, 10% glycerol, 1% BSA) at a ratio 1:10 at − 20 °C till use. After calibrating samples to room temperature, 12.5 ul of samples were transferred into light grey half-area Alpha plate-96 and followed with 12.5 ul of LDH detection enzyme mix with reductase substrate. Every plate contained a calibration LDH curve as control of linear range. The bioluminescence was measured by PHERAstar after 30 min incubation at RT. Data from both methods were collected from three experiments and analyzed with one-way ANOVA/Multiple comparisons for ATP assay and two-way ANOVA/Bonferroni test for LDH assay.

### Immunocytochemistry and proximity ligation assay

The treated primary hippocampal neurons were washed once with cold PBS and fixed at 21DIV with 4% PFA (Sigma) or 100% ice-cold methanol for 15 min [66]. In cultures inoculated with heparin-induced K18-tau fibrils, cells were fixed with 4% PFA with 1% Triton-X100 [21, 30, 31]. After three wash steps with PBS, PFA-fixed cells were permeabilized with 0.1% Triton in PBS and all blocked with 10% normal goat serum (NGS, ThermoFisher Scientific) and 1% casein in PBS. The plates were incubated with primary antibodies (Table 1) in PBS with 3% NGS overnight at 4 °C. Wells were washed 5 min three times with PBS and secondary antibodies were added for 1 h at 37 °C. The cells inoculated with human AD-tau samples were washed for 5 min 3 times with PBS, incubated with TrueBlack Plus Lipofuscin Autofluorescence Quencher in PBS (Biotium, 50ul/well) 10 min at dark, and washed three times with PBS. Cells were mounted with Fluoromount-G with DAPI (Invitrogen) and covered with 5 mm cover glass (Electron Microscopy Sci).

**Table 1:** Antibodies applied

Antibody	Isotype	Company, Cat #	Concentration
Primary antibodies			
Anti-mouse Tau (Clone: RTM47)	Rat, monoclonal	FUJIFILM Wako, 012–26963	1:1000 (IHC), 1:3000 (WB)
Anti-human Tau (Clone: RTM49)	Rat, monoclonal	FUJIFILM Wako, 015–26953	1:1000 (IHC)
Anti-Tau (316–355 aa, clone: 77G7)	Mouse, monoclonal	Biolegend, 816701	0.4 µg/ml (WB), (CDI, CSA)
Anti-Human PHF-Tau (clone: AT8)	Mouse, monoclonal	ThermoFisher, MN1020	1:400 (IHC), 1:1000 (WB)
Anti-Tau, x-421 (TauC3)	Mouse, monoclonal	BioLegend, 806304	1:400 (IHC)
Anti-3R Tau	Rat, monoclonal	FUJIFILM Wako, 016–26581	1:4000 (WB)
Anti-RD3 Tau (clone: 8E6/C11)	Mouse, monoclonal	Gift from Dr. R. de Silva	(CDI)
MAP2	Rabbit, polyclonal	Synaptic systems, 188002	1:1000 (IHC)
Homer 1/2/3	Rabbit, polyclonal	Synaptic systems, 160103	1:500 (IHC), 1:2000 (WB)
Bassoon	Chicken, polyclonal	Synaptic systems, 141016	1:500 (IHC)
Bassoon	Rabbit, monoclonal	Cell signaling, D63B6	1:3200 (WB)
Synaptophysin 1/2	Mouse, monoclonal	Synaptic systems, 101111	1:2000 (WB)
PSD95	Rabbit, polyclonal	Invitrogen, PA5-85783	1:2000 (WB)
GAPDH (HRP Conjugate, 14C10)	Rabbit, monoclonal	Cell signaling, 50-190-709	1:20,000 (WB)
Secondary Antibodies			
Anti-Rabbit IgG (H + L)/Alexa Flour 488	Goat, polyclonal	Invitrogen, A11008	1:500 (IHC)
Anti-Rabbit IgG (H + L) / Alexa Flour 633	Goat, polyclonal	Invitrogen, A21070	1:500 (IHC)
Anti-Rat IgG (H + L)/Alexa Flour 633	Goat, polyclonal	Invitrogen, A21094	1:500 (IHC)
Anti-Chicken IgY (H + L)/Alexa Fluor 633	Goat, polyclonal	Invitrogen, A21103	1:500 (IHC)
HRP anti-Rat IgG (clone: Poly4054)	Goat, polyclonal	BioLegend, 405405	1:3000 (WB)
Anti-mouse IgG/HRP	Sheep, polyclonal	Amersham, NA931-1ML	1:3000 (WB)
Anti-rabbit IgG/HRP	Donkey, polyclonal	Amersham, NA934-1ML	1:3000 (WB)

*IHC* immunohistochemistry, *WB* Western blot, *CDI* conformation-dependent immunoassay, *CSA* conformational stability assay

The proximity ligation assay was performed according to the manufacturer’s instructions (DuoLink) and [67, 68]. The PLA probes anti-rat/MINUS and anti-rat/PLUS IgG were prepared by conjugation of Duolink PLA PLUS and MINUS oligonucleotides and goat anti-rat IgG (H + L) secondary antibody (Invitrogen, #31220) in conjugation buffer overnight at RT. The reaction was halted by adding Stop Reagent and the anti-rat IgG PLA oligo-conjugated antibodies (PLUS and MINUS) were stored in a Storage Solution at 4 °C. Neurons were fixed with 100% ice-cold methanol for 15 min on ice, washed three times with PBS, and incubated with Duolink Blocking solution for 60 min at 37 °C. Incubation with primary antibodies, rat monoclonal anti-mouse tau and rabbit MAP2, in antibody diluent solution was overnight at 4 °C. Cells were washed three times for 5 min with wash buffer A and a mixture of anti-rat PLA oligoconjugated antibodies (PLUS and MINUS) was added for 1 h at 37 °C followed by three 5 min washing steps with wash buffer A. Ligation and amplification-polymerase reactions with washing steps were proceeded according to the instructions. After the PLA, cells were incubated with anti-rabbit IgG/AF633 antibodies for 1 h at 37 °C and washed three times for 5 min with PBS. Finally, the post-treatment with TrueBlack Plus Lipofuscin Autofluorescence Quencher followed as described above. The neurons were mounted with Duolink In Situ Mounting medium with DAPI covered with 5 mm cover glass. Confocal images were acquired with Leica HyVolution SP8 confocal microscope, objective 40x/oil with z-stacks of 0.35 µm. All inoculation experiments were performed as three independent experiments.

### Cell lyses

Cortical neurons in 12-well plates were washed twice with ice-cold PBS, lysed with ice-cold cell lyse buffer containing 1% Sarkosyl, a cocktail of protein proteases and phosphatases in PBS, and scraped (six wells for one treatment together in 300ul cell lyse buffer). Lysed cells were gently mechanically pressed through a syringe with a 25-gauge needle ten times, sonicated 3 × 5 s. each at 80% power with Sonicator 4000, and pellets were collected at 14,000 rpm at 4 °C for 30 min in Allegra X-22R tabletop centrifuge. Supernatants were transferred and stored at − 80 °C till further applications. Pellets were dissolved in cell lyse buffer, centrifuged under the same conditions one more time. The supernatant was discarded, and pellets were dissolved in 150 ul cell lyse buffer, and analyzed immediately by sandwich-formatted CDI of pellets described above, and western blots. These cells lyse fractions were collected from three independent experiments.

### Immunoassays

Western blots (WBs) were performed on pellets (one-third of the dissolved pellet in cell lyse buffer, which was 50 µl, was spun under the same conditions, and was dissolved in electrophoresis sample buffer), and supernatants from cell lyse of inoculated cortical neurons (12 µg protein/well) with antibodies against synaptic markers and mouse tau, respectively; cell lyses of cortical neurons (2 µg protein/well) with 3R-tau antibody to confirm the 3R/4R-tau evolution trend in neuronal cultures; and AD5-tau inoculated before (100 ng tau/well) and after combined immunodepleting with 77G7 and AT8 tau antibodies. The western blots were performed as described previously [17]. The details of primary and secondary antibodies are in Table 1. The incubation with primary antibodies was 2 h at RT, except for mouse tau antibodies to develop membranes of cell lyse pellets that were incubated overnight at 4 °C. The PVDF membranes were always developed with PonceauS (Millipore Sigma) after transfer and GAPDH loading control was applied after target protein WBs.

### Image and data processing

Microscopic and western blot images were processed with ImageJ software (https://imagej.nih.gov/ij/). GraphPad Prism 8 and Origin software were applied for graphs and statistics.

Confocal images are shown as max intensities of 0.35 µm z-stacks. The aggregated mouse tau quantification was performed from at least two independent experiments with multiple wells. The quantification of tau aggregates and MAP2 present in the tau aggregated areas was performed from 4 × 4 µm areas defined as regions of interests (ROIs). The plot profiles for grey values were obtained for both channels, red for mouse Tau and green for MAP2, in the ROIs. Maximum grey value from the plot profiles for each channel were applied for violin plots and one-way ANOVA and Bonferroni test. To evaluate the effect of inoculum concentration on levels of newly generated mouse tau aggregates, the auto threshold (MaxEntropy method) was applied for image of z-stacks maximum intensity projection. The number of particles (size 0.1-infinity µm^2) from the binary image of red channel (mouse tau) per slide was obtained. We expressed the data as x-fold increase of mouse tau aggregates to untreated cells (medium only, 0 ng of AD1-tau) and one-way ANOVA was performed. Quantification of PLA signal corresponding to mouse tau aggregates was performed from max intensity images. The number of particles in the size range of 0.4 µm—infinity was counted from 0 to 40 threshold binary images and normalized to the number of nuclei. One-way ANOVA with Bonferroni test was performed. The levels of synapses in primary neurons were quantified as the overlap signal for Bassoon (presynaptic marker, red channel) and Homer (postsynaptic marker, green channel). The color threshold plots of RGB images combined red and green channels were used for colocalization. The threshold was set as a scale of 0–255 for the total signal area (red, green, and overlapped combined) and 37–48 window for merged of green and red signal to yellow spectrum. The percentage of colocalization was calculated as a ratio of the merged signal area to the total signal area and multiplied by 100. One-way ANOVA/Multiple comparisons were performed as a statistic test.

Corrected total cell fluorescence (CTCF) of individual neurons was applied for quantification of Homer and Bassoon proteins in two independent experiments. The CTCF of Homer and Bassoon signals were calculated according to the following equation: CTCF = Integrated density—(area of selected cell × mean fluorescence of background readings) [67, 69]. Raw CTCF data were normalized to medium control cultures set to 1.0. One-way ANOVA performed with GraphPad Prism software (San Diego, CA, USA) was used for analysis of differences in normalized CTCF levels among AD-tau treated neurons. Data are presented as mean ± SD.

The presence of newly formed HMW mouse tau species in pellets of cell lyses from inoculated cortical neurons was evaluated as a density of the well column with MW > 50 kDa in the PVDF membrane normalized to PonceauS staining. One-way ANOVA of data obtained from triplets of 1 h and 14 days of samples’ inoculation was performed. The semi-quantification of synaptic protein levels on WBs of cell lyses supernatants obtained from inoculated cortical neurons was expressed as a normalized experimental signal calculated as the density of the observed experimental signal of the target protein divided by the lane normalization factor. The lane normalization factor was calculated as the observed signal of housekeeping protein (GAPDH) for each lane divided by the highest observed signal of GAPDH on the blot. These normalized experimental signals for specific pre- and post-synaptic proteins were averaged from three independent experiments and expressed as x-fold value normalized to medium-treated samples, the linear regression of mean ± SEM for each time point of all inoculums combined was performed to evaluate the correlation between time point and target protein levels.

## Results

### Conformational diversity of misfolded tau in PTA-enriched Sarkosyl-insoluble tau samples from human AD cases

For this study, we selected Sarkosyl-insoluble-tau inocula extracted from frontal cortex of six neuropathologically confirmed AD cases with sodium phosphotungstate [17, 51]. We determined the tau concentration and degree of misfolding by conformation-dependent immunoassay (CDI) and conformational stability assay (CSA) [17, 27]. Mouse tau (moTau) from wild-type mice C57Bl6/Tac was collected with the same experimental protocol and served as a normal tau negative control. Demographics and clinicopathological characteristics of AD cases are described in Table 2 and the detailed characteristics of AD-tau and moTau inoculation samples in Table 3.

**Table 2:** Demographics and clinicopathological characteristics of AD cases

In cell assays	Sex	Age	Disease duration	PMI	Neuropathological classification	APOE	
Units	F/M	Years	Month	Hours	A/B/C	Alleles	
AD 1	M	56	112	13	3/3/3	E3	E3
AD 2	F	71	15	53	1/3/3	E4	E4
AD 3	F	86	4	5	2/3/2	E3	E3
AD 4	M	82	60	28	3/3/2	E3	E4
AD 5	F	67	72	12	3/3/2	E3	E3
AD 6	M	76	60	20	3/3/3	E3	E4

**Table 3:** Concentration and conformational characteristics of Tau isoforms in AD cases

AD case	Total soluble tau	Insoluble total tau		Insoluble 3R tau		Insoluble 4R tau		3R/4R tau ratio	
Units	µg/ml	µg/ml	CDI D/N ratio	µg/ml	CDI D/N ratio	µg/ml	CDI D/N ratio	3R (%)	4R (%)
AD 1	10.9	9.2	73.8	1.0	32.2	8.2	87.7	11.0	89.0
AD 2	8.3	4.1	50.1	0.6	17.1	3.6	72.6	13.8	86.2
AD 3	16.0	10.1	39.4	1.3	20.9	8.7	45.6	13.3	86.7
AD 4	9.5	5.1	49.5	0.6	24.2	4.5	57.5	11.8	88.2
AD 5	8.5	15.1	34.4	1.8	42.4	13.3	33.5	11.9	88.1
AD 6	5.4	1.3	13.6	0.3	6.0	1.0	21.1	21.8	78.2
Mo C57Bl	15.1	0.094	0.7	0.001	0.8	0.093	0.7	1.6	98.4

The concentrations of Sarkosyl-soluble and insoluble tau were obtained with CDI in duplicate measurements and are expressed in µg/ml of 10% brain homogenate

To evaluate AD-tau samples, we applied conformational-dependent immunoassay (CDI) and conformational stability assay (CSA) adapted to measure stability profiles and conformational signatures of tau [17] (Fig. 1). The advantage of both CDI and CSA is to operate with preserved profiles of original tau conformers extracted from human AD brain tissue with minimal undesirable artificial modifications since these assays are independent of the absolute concentrations of misfolded tau protein and does not require in vitro amplification step or additional purification of PTA-extracts [35, 49]. The assays apply europium-labeled detection 77G7 tau antibody or 3R-tau antibody and capture DA9 tau antibody, which bind to linear epitopes in unfolded conformers of monomeric tau [27] outside of regions of pathological phosphorylation and acetylated amino acids.

**Fig. 2:**
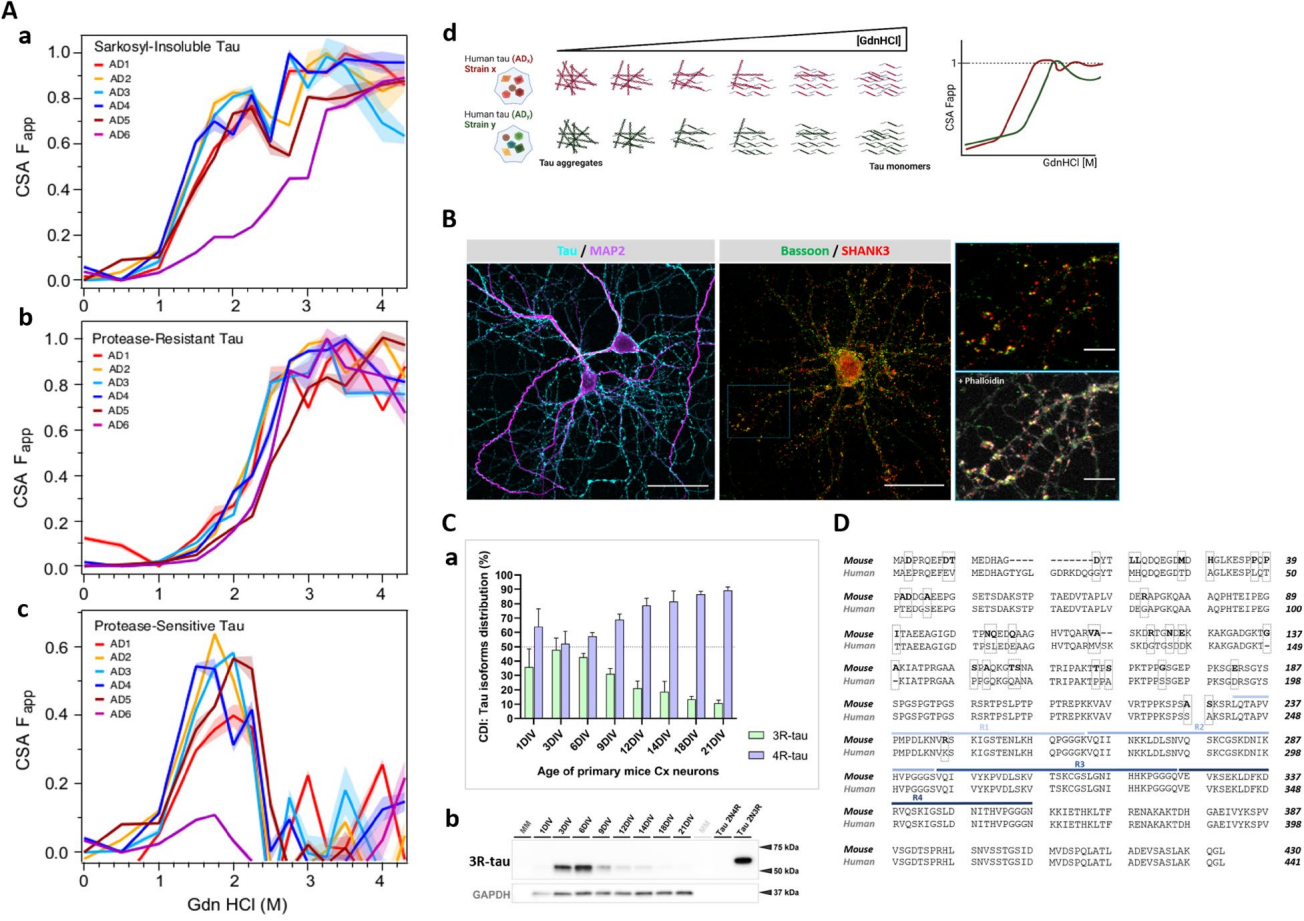
**A** Conformational stability assay (CSA) of sarkosyl-insoluble tau from AD brains applied as inoculum for neuronal cultures. Conformational profiles of human tau with incremental guanidine hydrochloride (Gdn HCl) concentrations to progressively unfold the misfolded tau to access epitope for interaction with 77G7 tau antibody (epitope 316–355 aa). AD-tau samples were **a** non-treated and **b** treated with Proteinase K before CSA, **c** the values of proteinase-resistant AD-tau were subtracted from non-treated AD-tau values. **d** Schematic representation of CSA. **B** Confocal images of primary neurons that we applied as the cell model. At 21DIV, primary neurons with distinguishable somatodendritic compartments (MAP2-positive, magenta) and axonal projections (Tau-positive, cyan). Mature primary neurons have functional synapses composed of pre- (Bassoon, green) and post-synaptic (SHANK3, red) compartments. Scale bars: 40 µm, cropped images: 10 µm. **C** The evolution of tau isoforms in primary cortical neuronal cultures: **a** the percentage of 3-repeat (3R) and 4-repeat (4R) tau isoforms in cell lysis of mouse cortical primary neurons in different days of cultures measured by conformation-dependent immunoassay (CDI) in three independent experiments. **b** Western blot of cell lysis of primary neurons in different days of cultures developed with 3R-specific tau antibody (clone: 2A1-1F4). **D** The amino acid sequence of mouse and human tau (2N4R isoform) varies in the N-terminal part of tau molecule, which is used in the production of mouse- and human-specific tau antibodies. Microtubule-binding domain composed of repeat domains (R1-R4) contains the pro-aggregation part of tau called paired helical filament (PHF) core and is identical in mouse and human tau

The CDI data obtained for native conformers of misfolded tau and fully denatured conformers by Gdn HCl of the same misfolded tau are used in calculating total tau levels and denatured/native (D/N) ratio—an overall different misfolding characteristic of tau conformers [17]. In CSA, the AD samples, non-treated or treated with proteinase K, are mixed with increasing concentrations of Gdn HCl to progressively unfold the misfolded tau, and the profiles of conformational stability curves describe the conformational signatures of misfolded tau strains in individual AD-tau samples (Fig. 2Ad) [70].

The data from CDI (Table 3) showed that all six AD cases vary in levels of Sarkosyl-insoluble tau and demonstrate differential conformational characteristics of misfolded tau expressed as D/N ratios. The contributions of 3R- and 4R-tau isoforms in Sarkosyl-insoluble fractions were 13.9 ± 3.99% and 86.1 ± 3.99%, respectively, which corresponds to results obtained previously [17]. The D/N ratios of 6.0–42.4 for 3R-tau and 21.1–87.7 for 4R-tau confirmed the diversity of misfolded tau conformers among individual human AD cases. Sarkosyl-insoluble tau extracted from AD cases yielded also complex CSA profiles with 50% of tau conformers unfolding (fractional change of unfolding (F_app_) values of 0.5) between 1.3 and 1.8 M Gdn HCl with exception of AD6 with 3 M Gdn HCl to reach 50% unfolding. The major peaks corresponding to high degree of unfolding occurred at ~ 2.2 M Gdn HCl followed by complete unfolding starting at ~ 2.8 M Gdn HCl, and the individual CSA profile varies above F_app_ value of 0.5 (Fig. 2Aa). The CSA profiles of protease-resistant core of Sarkosyl-insoluble tau (proteinase K treated) shifted the 50% threshold of misfolded tau conformers’ unfolding to the range between 2.1 and 2.6 M Gdn HCl (Fig. 2Ab). The CSA unfolding profiles of protease-sensitive misfolded tau were obtained by subtracting the individual curves of protease-resistant tau from those of total Sarkosyl-insoluble tau (Fig. 2Ac). Interestingly, the dip at ~ 2.5 M Gdn HCl for AD1-5 tau in Sarkosyl-insoluble total tau (Fig. 2Aa) distinct subsets of conformers we observed previously in larger AD cohort [17]. Based on both CDI data and CSA measurements, we concluded that Sarkosyl-insoluble tau aggregates in human AD tau samples used in this study encompass a spectrum of distinct conformers within each individual sample and variable fraction of misfolded protease-sensitive tau.

### Primary neurons from wild-type mice applied for modeling of misfolded tau conformers’ diversity

To monitor the effect conformational diversity of human AD-tau samples on synapses, we established cortical and hippocampal primary neurons isolated from wt mice as a cellular model. The primary neurons at 21DIV in cultures exhibit properties of mature neurons with axonal network separated from the somato-dendritic compartment and synaptic spines with synapses where pre- and post-synaptic protein markers (Bassoon and SHANK3, respectively) partially colocalized (Fig. 2Ba). The advantage of this cellular model is that neurons express physiological levels of mouse neuronal tau, and when exposed to human AD tau, the induced aggregation of mouse tau can be easily distinguished from human AD tau with mouse tau-specific antibodies with epitopes at the tau N-terminus (Fig. 2D) [71].

The isoform components of misfolded tau conformers vary in different tauopathies [72], and even though AD belongs to 3R/4R secondary tauopathy [73], the 3R- and 4R-tau isoforms are not equally represented in misfolded tau aggregates from human AD brains, of which the content of 4R-isoforms is approximately four-times higher than 3R-tau isoforms in misfolded tau conformers (Table 3) [17]. To our best knowledge, this is the very first study where the percentages of 3R and 4R isoforms in mouse primary neurons were directly monitored by CDI during the neuronal maturation; approximately equal content of both isoforms was observed in cultures in the early stages (3DIV-6DIV) and then the gradual increase of 4R isoform (Fig. 2Ca) paired with the decrease of 3R isoform from 9DIV was observed, and these trends were confirmed by WB (Fig. 2Cb).

### Human AD-tau inoculation of primary neurons causes minimal cytotoxicity effect and does not affect cell viability

Before we started to evaluate the effects of AD-tau strains on mouse tau and synapses in primary neurons, we first investigated the cytotoxic effect of AD-tau and control samples on primary neurons and their post-treatment viability at a concentration working range in 96-well plate format. The cell viability measured as ATP levels was minimally affected by AD-tau treatment (Additional file 1: Fig S1A). Compared to untreated cells (medium), cultures inoculated with samples AD3-tau and AD5-tau had decreased ATP levels to 80% (p < 0.05), but compared to 0.2% Triton treatment mimicking cell death, where the production of ATP levels is close to 0% (p < 0.0001), the vast majority of neurons treated with AD-tau samples at the highest concentration 45 ng is viable. The exception is AD6-tau inoculum, when applied in concentration of 45 ng, the neurons died (p < 0.0001), and thus we decided that we will apply 3 time less tau concentrations with AD6-tau sample. We believe that the toxic effect of AD6-tau in higher tau concentrations is related to need of high volume of AD6-tau inoculum as it contains lower levels of insoluble tau in brain tissue, thus some tracer impurities co-extracted with Sarkosyl-insoluble tau might cause toxicity effect in higher dose (Table 3). But we also do not exclude the possibility of presence of tau strains toxic to cells [34] in AD6-tau inoculum. There were no statistically significant variations in lactate dehydrogenase (LDH) activity in cells medium harvested from wells before treatment (p = 0.659) (Additional file 1: Fig S1Bb), and AD-tau treatment for 14 days shows only minimal cytotoxicity effect of AD-tau material, where the activity of LDH was utmost 1.5 times elevated compared to non-treated cells in medium. Two-way ANOVA shown that effect of inoculum applied (p < 0.001) but no effect regarding the AD-tau concentration (p = 0.444), Bonferroni test indicates statistical differences with cells inoculated with AD1-tau and mTau (both p < 0.001) and AD2-tau (p < 0.05) (Additional file 1: Fig S1Ba). Wells treated with 0.2% Triton-X100 causing cell death were used as controls, the ATP production was halted and LDH activity in medium increased four times compared to non-treated cells. Thus, we decided to inoculate cells with concentrations 45 ng AD-tau and the corresponding volume of control mTau samples per 5*10^3^ seeding cells, and 180 ng of tau per 2.7*10^5^ cells, in the case of AD6-tau the concentrations were set as 15 and 60 ng, respectively.

Cumulatively, the data indicate that the range of concentration of AD-tau inocula we applied had minimal direct cytotoxicity effect and the cell viability sustained, thus we could monitor and evaluate propagation of tau misfolding and its effect on synapses over time.

### Human AD tau conformers trigger aggregation of endogenous mouse tau in wild-type primary neurons

**Fig. 3:**
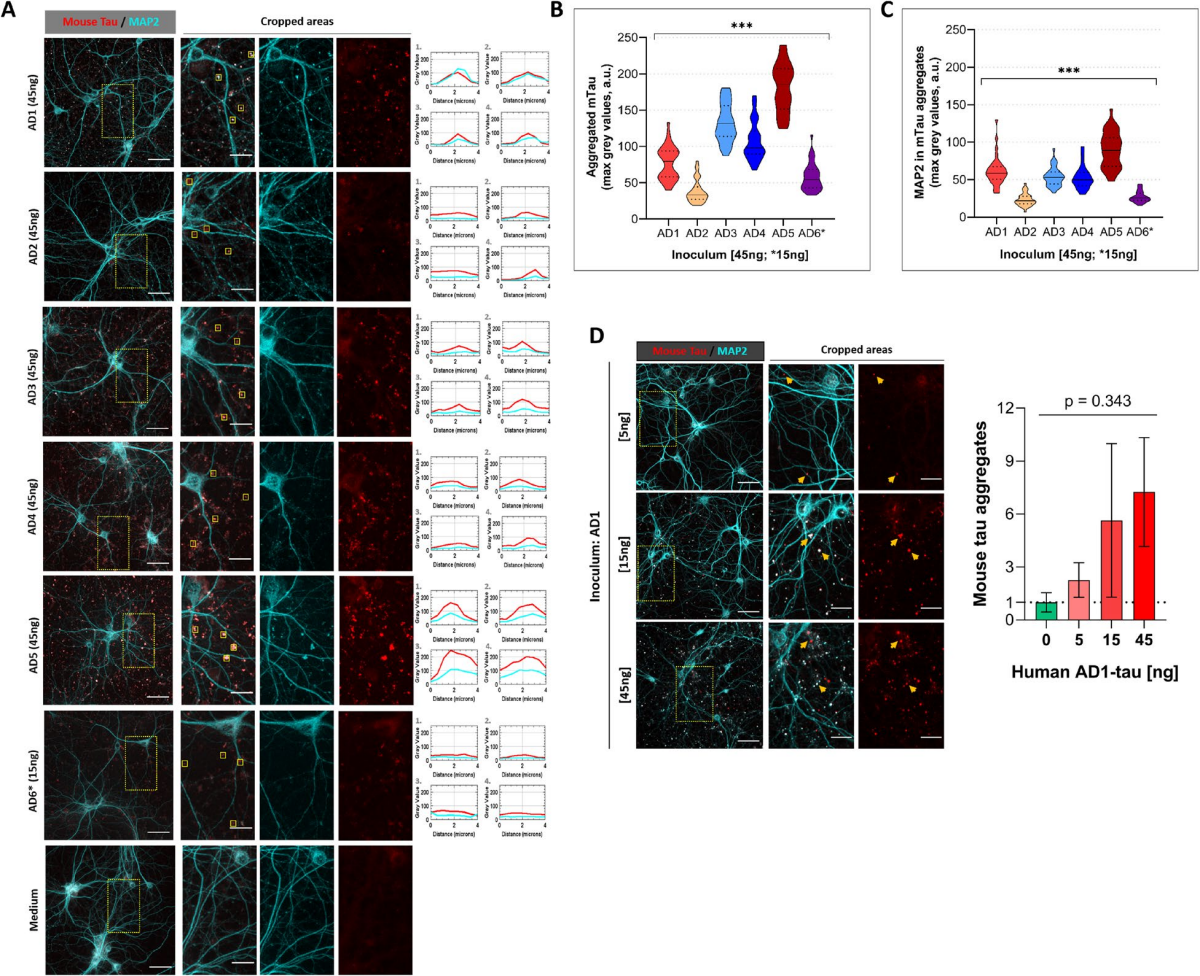
Representative confocal microscopy images of 21DIV primary hippocampal neurons inoculated with AD-tau samples at 7DIV for 14 days. **A** All six AD-tau inoculates (AD1-6) trigger misfolding/aggregation of endogenous mouse tau to various degrees with different fluorescent intensities and sizes of the fluorescence signal (red), MAP2 (somatodendritic marker, cyan) is present to some extend in mouse tau-positive aggregates. The pattern of tau aggregation is different in an inoculum-to-inoculum manner. Squares of 4 × 4 µm were applied as regions of interest in areas of profoundly aggregated tau and the plot profiles for mouse tau (red) and MAP2 (cyan) are displayed. **B**, **C** Violin plots of maximum grey values from plot profiles of 4 × 4 µm areas for aggregated mouse tau (B) and MAP2-positive signal in areas with aggregated tau (C), mean ± SEM. One-way ANOVA showed statistical significance in the maximum intensity of mouse tau aggregates and MAP2 fluorescence present in these aggregates’ areas across all six inoculum-treated neurons (*** p < 0.0001). Bonferroni test in the graph (B) confirmed significant variability among all six inocula to trigger tau aggregation (*** p < 0.0001, except AD1 vs AD6: *p = 0.0163, AD2 vs AD6: *p = 0.0083). The occurrence of MAP2 in tau aggregates greatly varied among most of the AD inocula (p < 0.0001, Bonferroni test), except among samples AD1 vs. AD3 (p = 0.768), AD1 vs. AD4 (p = 0.361), AD3 vs. AD4 (p = 1), and AD2 vs. AD6 (p = 1). The max intensity values of plot profiles of 4 × 4 µm ROIs were averaged from two independent experiments (ROIs, n = 36 per inoculum). **D** Images of neuronal cultures inoculated for 14 days with AD1-tau sample of three different concentrations (5, 15, and 45 ng/well) show that the degree of mouse tau aggregation is inoculum concentration-dependent. Graph representing x-fold increase of mouse tau aggregates as number of particles (0.1-infinite µm size) to untreated cells (medium only, 0 ng of AD1-tau). One-way ANOVA did not show statistical significance (p = 0.343) due to high variability, mean ± SEM. Values were collected from confocal images of two independent experiments (n = 15). All images are presented as maximum intensities of 35 z-stacks (0.35 µm each), scale bars: 50 µm of large images, and 20 µm of cropped areas. The staining for aggregated tau was performed after ice-cold methanol fixation

**Fig. 4:**
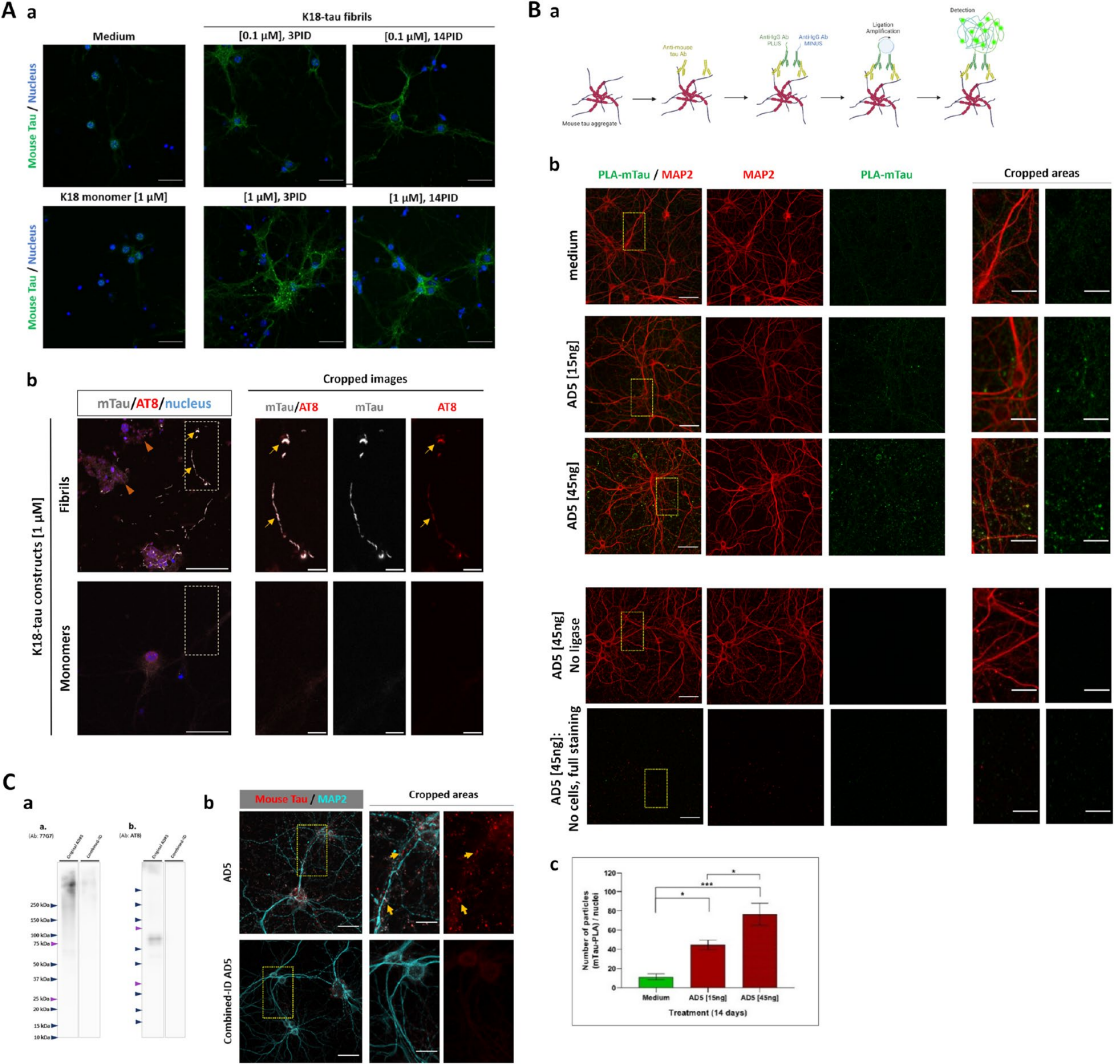
Additional approaches to confirm the detection of aggregated mouse tau in primary neuronal cultures by application of artificial K18-tau fibrils, proximity ligation assay (PLA) and tau-immunodepleted AD5 sample. **A** Primary hippocampal neurons were treated with **a** K18-tau fibrils in concentrations of 0.1 and 1 µM at 7DIV and 18DIV, inoculated for 14 days (14PID) and for 3 days (3PID), respectively. Cultures were fixed with 4% PFA and 1% Triton-X100 to extract soluble tau. Compared to controls (cells treated with medium only), the K18-tau fibrils-inoculated cultures show the presence of insoluble mouse tau in an inoculum dose-dependent manner. In **b** neurons were treated at 7DIV with 1 µM of K-18-tau monomers or fibrils and fixed with 4% PFA and 1% Triton-X100 at 21DIV. In K18-tau fibrils treated neurons, insoluble tau colocalized with AT8-positive staining in projections (yellow arrows). Pathologically insoluble tau (AT8-positive) is also observed around nucleus in more diffuse manner (orange arrowheads). Compared to K18-tau monomers’ treatment, the presence of K18-tau fibrils contributes to conversion of endogenous mouse tau into more pathological tau species. **B** In situ proximity ligation assay (PLA). **a** Schematic representation of PLA experiment for detection of aggregated mouse tau. In mouse tau aggregates, tau molecules are in proximity and can be captured by mouse-specific tau antibodies bound to the N-terminal part of tau. These antibodies are then recognized by secondary anti-IgG PLUS/MINUS PLA probes, which are conjugated with short complementary oligonucleotide sequences with a fluorophore. The oligonucleotides undergo ligation and amplification in situ to increase the sensitivity of the fluorescent signal. **b** PLA images of mouse tau in ice-cold methanol fixated cultures (21DIV) inoculated for 14 days with two different concentrations (15 and 45 ng) of AD5 samples demonstrate that fluorescent signal (green) is specific to the occurrence of aggregated mouse tau. Controls of full PLA staining of AD3-inoculated wells with no cells and AD3-inoculated cell cultures following the PLA staining protocol without ligase confirm that PLA signal is specific to aggregated mouse tau. **c** One-way ANOVA was applied to number of particles per nuclei from six images per treatment and showed statistical significance (*** p < 0.001, n = 6), and Bonferroni test also confirmed the inoculum concentration-dependent aggregation of mouse tau (medium vs. 15 ng: *p = 0.0235, medium vs. 45 ng: ***p = 0.0001, 15 ng vs. 45 ng of AD5-tau, *p = 0.0219). **C** Immunodepleted AD5-tau sample as a control. **a** Western blot: AD5 tau-immunodepleted (ID) of sarkosyl-insoluble human tau by combined AT8 and Tau5 antibody immunomagnetic separation shows a significant decrease of HMW tau and lack of AT8 tau compared to the original AD5 sample. **b** Primary neurons treated with combined-ID AD5 sample show no aggregated mouse tau after 14 days of inoculation whereas aggregated mouse tau signal is detected in the cultures treated with the original AD5-tau sample. The images are presented as maximum intensities of 35 z-stacks (0.35 µm each), scale bars: 50 µm of large images, 20 µm of cropped areas. The staining for aggregated tau was performed after ice-cold methanol fixation

The presence of various misfolded tau conformers in individual AD cases has been now documented with various approaches [17, 21] and our goal was to investigate their seeding, propagation, and synaptic effects in wt mouse primary neurons and establish a prion-like cell model. Six PTA-extracted Sarkosyl-insoluble human AD-tau samples (AD-tau samples) were applied in this study (Table 3). The hippocampal neurons were inoculated at 7DIV for 14 days with AD-tau samples followed by ice-cold methanol fixation, which extracts soluble tau molecules [66], and a mouse tau-specific antibody was applied. All six applied AD-tau inocula trigger endogenous mouse tau aggregation with distinct unique patterns (Fig. 3A). The mouse tau misfolding is dependent on the presence of misfolded human tau conformers, as control cultures in the medium do not show mouse tau aggregates (Fig. 3A), and even the neurons treated with AD-tau immunodepleted with combined Tau5/AT8 immunosorbent show no significant mouse tau aggregates compared to the original AD-tau inoculum applied (Fig. 4C). Moreover, the degree of mouse tau aggregation is likely to be concentration dependent as shown with AD1-tau inoculum in three concentrations 5, 15, and 45 ng/well, respectively (Fig. 3D). The tau forms truncated at D421 or phosphorylated at S202/T205 that are linked to tau pathology and formation of PHFs in AD [74–77] (Additional file 1: Fig S2) were present only in AD-tau inoculated cultures (p < 0.0001, Additional file 1: Fig S2Aa, b). Phosphorylation at residues S202/205 detected with AT8 antibodies was present in cultures containing primarily insoluble forms of tau (ice-cold 100% methanol fixation), with fluorescent signal partially overlapping with residual signal of AD tau inoculum still present at 21DIV (Additional file 1: Fig S2B). Notably, the TauC3 truncated species were observed only when classic fixation with 4% paraformaldehyde was applied (Additional file 1: Fig S2Aa). Whether these differences are due stages in tau misfolding and aggregation or separate pathways for different tau conformers [75] will require a systematic mass spectrometry study. Control staining conducted (i) on wells with no seeded cells followed full treatment and staining as described in Methods, and (ii) on wells with inoculated cells stained following the protocol without primary antibodies showed that staining with mouse-specific tau antibody is specific to aggregated mouse tau in methanol fixed samples with no observed nonspecific signal and the setting for image acquisition was set with minimal non-specific fluorescence signal. The fully treated and stained control wells with unseeded cells were critical controls to evaluate specificity of all primary antibodies, backgrounds in PLD-coated plates and to control for residual autofluorescence of lipofuscin or other compounds [78–81] present in the AD brain-derived tau inoculum (Additional file 1: Fig S3A). Additionally, at the tested concentrations of inoculum, we optimized protocols with an additional quenching step with TrueBlack Plus Lipofuscin Autofluorescence Quencher (data not shown). As a result, with applied mTau (wild-type mice) inoculum, after 14 days we observed minimal background tau aggregation signal and well-preserved MAP2 projections comparable to untreated cells (Additional file 1: Fig S3B).

Parallel with the applied methanol fixation and immunostaining approach, we conducted independent experiments with K18-fibrils and PLA method to confirm the occurrence of aggregation events of endogenous mouse tau in wt primary neurons (Fig. 4A, B). Mouse tau aggregates were present after treatment with artificial misfolded K18 tau constructs followed with 0.1% TritonX-100 in 4% PFA fixation (Fig. 4A) applied to wash out soluble tau molecules as previously described in [21, 30]. Moreover, AT8-positive tau occurs after 14 days of inoculation with K18-tau fibrils, showing partial colocalization with insoluble mouse tau and a more diffuse pattern in soma (Fig. 4Ab), which provides direct evidence for K18-fibrils triggered evolution of pathologically phosphorylated tau (pS202/pT205) linked to formation of PHFs [76]. Proximity ligation assay (PLA) is a technology enabling the visualization of two target proteins in high proximity (< 40 nm) [82, 83], thus we separately linked mouse-specific tau antibodies with a set of two complementary DNA oligomers, which will follow hybridization step and PCR amplification with fluorescent probes when they are in sufficient proximity (Fig. 4Ba) [83]. The number of fluorescent PLA dots was counted for two concentrations of AD5-tau inoculum and medium-treated cells and normalized for the number of nuclei per frame. The PLA means were significantly different (p < 0.001) and compared individual groups with the Bonferroni test showed statistical difference (medium vs 15 ng AD5-tau: p < 0.05; medium vs 45 ng AD5-tau: p < 0.001; 15 ng vs 45 ng AD5-tau: p < 0.05) confirming the inoculum concentration-dependent aggregation of mouse tau (Fig. 4Bb). PLA controls stained without primary antibodies and performed without the ligase were negative for PLA dot signal (Fig. 4B).

We concluded from these observations that the aggregation of endogenous mouse tau in wt primary neurons is triggered in dose-dependent manner by human misfolded AD-tau conformers and also by recombinant K18 tau fibrils, and that various effects obtained at the same concentration reflect intrinsic characteristics of distinct AD isolates.

### Conformation diversity of human AD tau results in various aggregation properties of endogenous mouse tau in primary neurons

Since the aggregation of mouse tau triggered with human AD-tau can be detected in wt primary neurons, we investigated the aggregation rates and kinetics of mouse tau aggregation linked to individual AD-tau inoculum. At the same concentration of the tau, the pattern of tau aggregation is different in an inoculum-to-inoculum manner. First, we quantitatively evaluated the aggregates’ properties from confocal images in two independent experiments where the cells were inoculated with AD-tau cases and observed for 14 days and then stained for aggregated mouse tau and dendritic marker MAP2 (Fig. 3A). Four areas were cropped from maximum intensities of z-stacked images for each case and applied for analyses of individual mouse tau-positive aggregates as 4 × 4 µm regions of interest outside of the dendritic projections. The grey value plots for both mTau and MAP2 channels were implemented, and the maximum plot values were used for data analysis by one-way ANOVA. Maximum signal values in aggregated areas for mouse tau and MAP2 were statistically different among AD-tau inocula (both p < 0.0001, Fig. 3B, C). Interestingly, various extent of colocalization of MAP2 and mouse tau aggregates might suggest inoculum-dependent alterations of dendritic integrity as well as the presence of varicosities of MAP2-positive dendritic projections (Fig. 3A) [84]. These results indicate that abnormal tau propagation initiated with brain tau conformers at nanograms’ scale induces alterations of dendritic integrity and synaptic loss in the absence of an overt neurodegeneration.

**Fig. 5:**
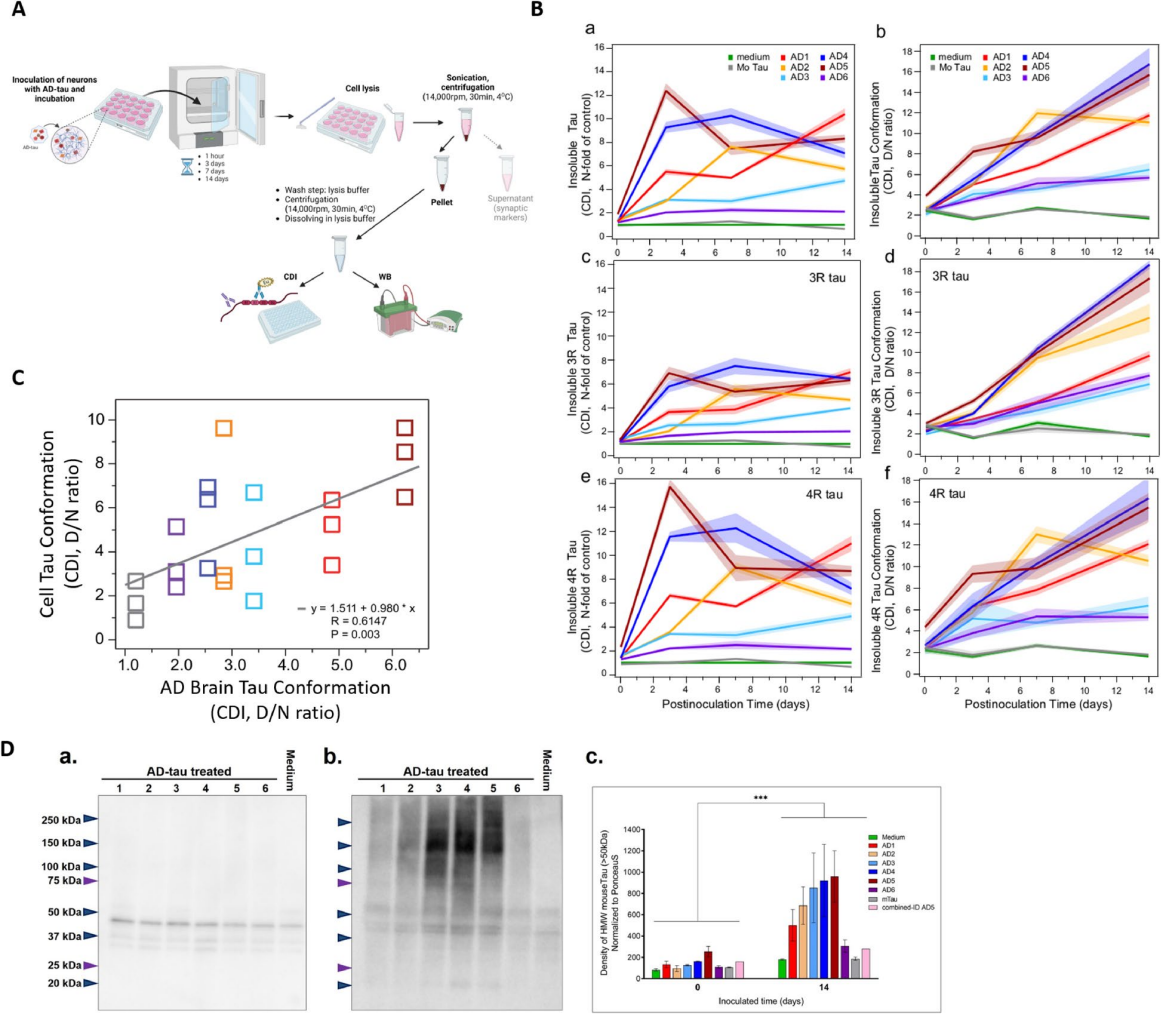
Rate of mouse tau aggregation and conformation of newly formed aggregates. **A** The experimental workflow for pellets containing sarkosyl-insoluble mouse tau obtained from cell lyses for analyses by conformation-dependent immunoassay (CDI) and western blots. **B** Kinetics of evolution of sarkosyl-insoluble mouse tau conformers in neuronal cultures: CDI data from cell lysis of cortical neurons inoculated by AD-tau 1–6 and mouse tau (Ctrl) samples at 7DIV for 1 h (starting point), 3, 7, and 14 days. N-fold relative sarkosyl-insoluble **a** total tau levels, **c** 3R-tau levels, and **e** 4R-tau levels in cell lysis inoculated with AD-tau 1–6 and mouse tau (control) to non-treated cultures (medium) show different kinetics of insoluble tau formation for individual AD-tau inoculates. The conformation of insoluble **b** total tau, **d** 3R-tau, and **f** 4R-tau in cultures expressed as D/N ratio from CDI data of native sarkosyl-insoluble cell lysis samples (native, N) and Gdn HCl-treated samples (denatured, D) show mostly increasing of mouse tau misfolding and the variability dependence on human AD-tau inoculates. All CDI graphs are means ± SEM (n = 6; three independent experiments with two values for CDI measurement. **C** Correlation of tau conformation in the original samples of human AD-tau (x-axis) with sarkosyl-insoluble tau newly formed in cortical neurons inoculated for three days with AD-tau (y-axis) expressed as D/N ratios from CDI shows linear regression with statistical significance (p = 0.003, n = 6; three independent experiments with two values from each CDI measurement). **D** Western blots of sarkosyl-insoluble fractions of cell lyse of primary neurons inoculated with AD-tau at 7DIV and **a** treated for 1 h and **b** 14 days before cell lyses developed by specific mouse tau antibody (clone RTM47) show various degrees of increase of high-molecular-weight (HMW) mouse tau conformers after 14 days of inoculation period compared to 1 h treatment. **c** Semi-quantification of the density of HMW tau signal (> 50 kDa) normalized to PonceauS staining by one-way ANOVA shows a statistically significant difference between the starting point and 14 days after inoculation (*** p < 0.001). All data are expressed as mean ± SEM combined from three independent experiments

In another approach to investigate the properties of accumulating tau aggregates, we inoculated cortical neurons and collected cell lysates at 4 time periods (1 h, 3d, 7d, and 14d, respectively) for western blots and CDI with no confounding purification or chemical processing steps (Fig. 5A). By applying CDI, we were able to evaluate the total insoluble tau concentrations and their conformations (D/N ratio) in sarkosyl-insoluble cell lysate fractions expressed as n-fold to medium-treated controls (Fig. 5B). The concentrations of insoluble tau from AD-tau-treated neuronal cell lysates raised significantly over time and demonstrated different aggregation kinetics for each individual AD-tau inoculum (Fig. 5Ba). The values of CDI conformational D/N ratios corresponding to insoluble tau (higher D/N ratio means more hidden epitopes in PHF core), indicate different sets of conformations of newly formed mouse tau aggregates induced by each AD-tau inoculum (Fig. 5Bb). The vast majority of insoluble tau consists of 4R tau (~ 80–90%, data not shown) and the shapes of curves for 4R tau D/N ratios (Fig. 5Bf) are parallel with the total tau D/N ratio curves (Fig. 5Bb). Control inoculation of neurons with volume-adjusted samples prepared from healthy C57Bl/6Tac mice with minimal content of sarkosyl-insoluble tau (mTau (Mo C57Bl), Table 3) show only residual levels of insoluble tau in cell lyses with the D/N ratio corresponding to the medium-treated controls (Fig. 5B, grey curves). We examined the correlation of D/N ratios (tau conformation state) of original AD brain tau inocula vs. newly misfolded cell tau. Surprisingly, in neurons treated for 3 days, the CDI D/N ratios of cell tau showed a highly significant correlation (p = 0.003) with the D/N ratios of AD-tau brain inocula, with some intra-individual range (Fig. 5C). We did not observe a statistically significant correlation in higher time points of treatment (7d and 14d; data not shown). Taken together, the newly forming tau aggregates in mice neurons initially copy the conformation of the original AD brain tau with high fidelity but then the different tau isolates start evolving independently, suggesting competitive template-misfolding with kinetic advantage of more aggressive prion-like conformers as was observed with human prions [85].

Western blots of sarkosyl-insoluble fractions of cell lysates were applied with mouse-specific tau antibody to confirm specific mouse tau aggregation in the presence of AD tau inoculum. The mouse tau signal was significantly higher in cultures treated with AD-tau samples for 14 days (Fig. 5Db) compared with 1 h (Fig. 5Da) (p < 0.001, Fig. 5Dc). Negative controls such as mTau and immunodepleted-AD5 samples have minimal or no effect on mouse tau aggregation (Fig. 5Dc, Additional file 1: Fig S4A). Moreover, the plotted curves from western blot data (Additional file 1: Fig S4B) showed similar trend as those in CDI (Fig. 5Ba). All these data were collected from three independent experiments.

These quantitative biophysical and imaging data indicate that wild-type 2N4R mouse tau expressed predominantly in matured cultured neurons can be misfolded and aggregated with various rates when exposed to human AD-tau and that individual AD-tau with distinct conformational characteristics trigger the formation of predominantly 4-four-repeat mouse tau conformers with homologous initial characteristics which are different from case to case. Moreover, misfolded tau conformational diversity occurring in individual AD cases [17, 21] is transmissible to newly misfolding mouse tau aggregates in the early stages of the process but the tau conformers evolve later independently. Whether some conformers have a higher propensity to trigger tau aggregation, thus leading the higher rate of misfolding processes in a templated manner with will require higher resolution tools such as hydroxylation footprinting and mass spectrometry [86].

### Misfolding of mouse tau affects levels of scaffolding proteins enriched in the post-synaptic terminal and decreases synaptic levels

**Fig. 6:**
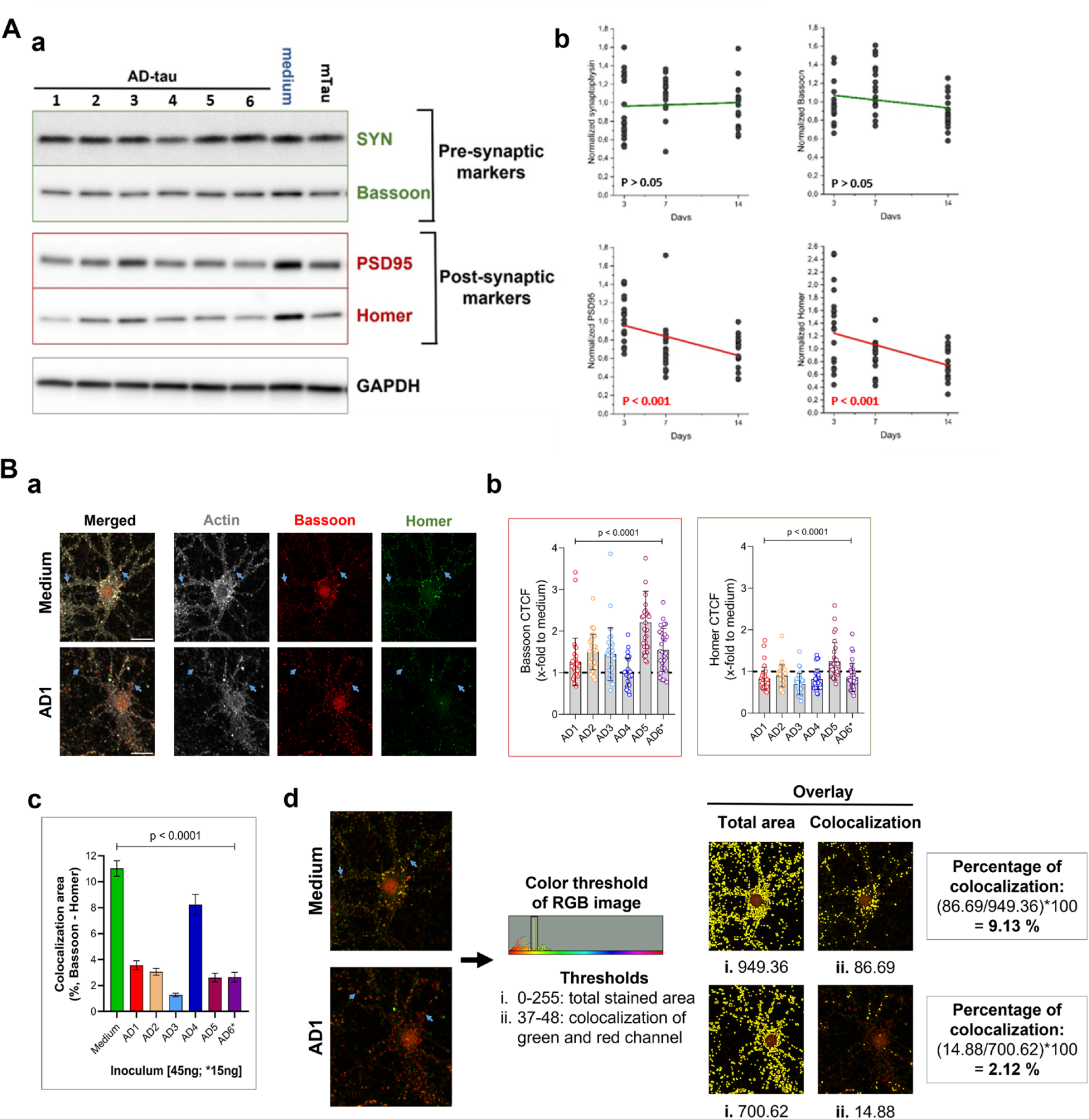
Effect of human AD-tau triggered endogenous mouse tau aggregation on synapses. **A** **a** Western blots of two pre-synaptic markers (synaptophysin (SYN) and Bassoon) and two post-synaptic markers (PSD95 and Homer) of lysed neurons after 3, 7, and 14 days of inoculation with AD1-6 samples and controls. **b** The linear regression indicates the overall effect on the post-synaptic compartment (p < 0.001, n = 18 per time point, three independent experiments for each AD-tau inoculum, six AD-tau samples). There is no statistically significant difference in pre-synaptic marker levels at different time points (p > 0.05, n = 18 per time point). The data were expressed as densities of target protein bands normalized to GAPDH and then as n-fold change to medium-treated cells and combined from three independent experiments. **B** The levels of synapses in primary neurons inoculated with AD-tau samples. **a** Representative images of maximum intensities from 0.35 µm z-stacks of control (medium) and AD1-inoculated culture stained for actin (grey), Bassoon (cyan), and Homer (magenta) display disruption in actin pattern staining, a slightly elevated number of Bassoon dots (red) and overall decreased fluorescence of Homer staining (green) in AD-tau treated cells. Scale bars: 30 µm. **b** Neurons treated with various AD-tau show statistically significant differences in levels of both markers, the presynaptic Bassoon and the postsynaptic Homer, expressed as a corrected total cell intensity (CTCF) in individual neurons. One-way ANOVA was applied (mean ± SD; p < 0.0001, n = 30–35 per AD-tau case summarized from two independent experiments). **c**, **d** The overlap of Homer and Bassoon signal was quantified by colocalization of the color threshold of RGB image combined from red and green channels and the threshold was set as a scale of 0–255 for the total signal area (red, green, and overlapped combined) and 37–48 window for merged of green and red signal to yellow spectrum. The percentage of colocalization was calculated as a ratio of the merged signal area to the total signal area and multiplied by 100. When compared to untreated neurons, cultures with aggregated mouse tau triggered by all AD-tau samples had significantly reduced levels of synapses (One-way ANOVA with multiple comparisons, p < 0.0001, n = 32 per treatment from two independent experiments)

To investigate the link between misfolding and aggregation of tau protein and synapses alterations [12–14], we monitored the levels of two pre-synaptic proteins (Synaptophysin and Bassoon) and two post-synaptic scaffolding proteins (PSD95 and Homer) by western blots in cell lysates of cortical neurons inoculated with AD-tau cases. The levels were monitored 3, 7, and 14 days after inoculation (Fig. 6Aa), the data were normalized to GAPDH and then to un-treated cultures. The normalized triplicate experiments were analyzed against time with linear regression. We observed no statistically significant trend for pre-synaptic markers (both p > 0.05) but we observed significantly time-dependent decreases in levels of both post-synaptic markers, PSD95 and Homer (both p < 0.001, Fig. 6Ab). The control cultures inoculated with Mo Tau and immunodepleted AD5-tau sample (combined-ID) displayed no significant correlation by linear regression either for pre-synaptic markers (Synaptophysin, p = 0.765; Bassoon, p = 0.203) or post-synaptic markers (PSD95, p = 0.283; Homer, p = 0.678; Additional file 1: Fig S5).

These highly reproducible time-course data indicate alterations in levels of post-synaptic scaffolding proteins as a result of endogenous mouse tau aggregation triggered by human AD-tau, and thus provide direct evidence for the degradation of post-synaptic compartment of synapses as the earliest event associated with the propagation of misfolded tau conformers. To further evaluate the effect of altered post-synaptic compartment triggered by tau misfolding spreading on synapse levels, hippocampal neuronal cultures inoculated with AD-tau 14 days earlier were fixed and stained with antibodies against bassoon (pre-synaptic marker) and Homer (post-synaptic marker) (Fig. 6B). Both synaptic proteins showed various changes in their density in cultures inoculated with distinct AD-tau (p < 0.0001; Fig. 6Bb). The presynaptically localized bassoon was slightly elevated in all AD-tau cases, except AD-tau #4, which was documented previously in rat model of tauopathy [9]. On contrary, we observed loss of postsynaptic marker Homer in AD-tau treated cultures, except AD-tau #5 (Fig. 6Bb), as observed with western blots’ data (Fig. 6Ab). Moreover, their colocalization reflects the presence of intact neuronal synapses [87]. The cultures treated with AD-tau showed significant decrease of colocalization areas compared with untreated cells (p < 0.0001, Fig. 6Bc). We calculated the percentage of colocalization area of manually cropped neurons based on actin staining by phalloidin (Fig. 6Ba). The RGB images were created by merging red (Bassoon) and green (Homer) channels and color threshold set in two ranges: 0–255 as an entire area of staining positive and 37–48 as yellow part of the RGB spectrum representing the overlap of red and green channel. The percentage of colocalization was expressed as percentage of overlap area from the total area (example shown in Fig. 6Bd). Interestingly, all AD-tau cases were associated with minimum three-fold decrease of the colocalization signal (p < 0.0001), except AD4-tau but the comparison with untreated cells was still significant (p < 0.0001). The actin disruption pattern can be seen in AD-tau treated neurons and the slight elevation of Bassoon signal and decrease of Homer-positive staining was an overall pattern observed (Fig. 6Ba).

**Fig. 7:**
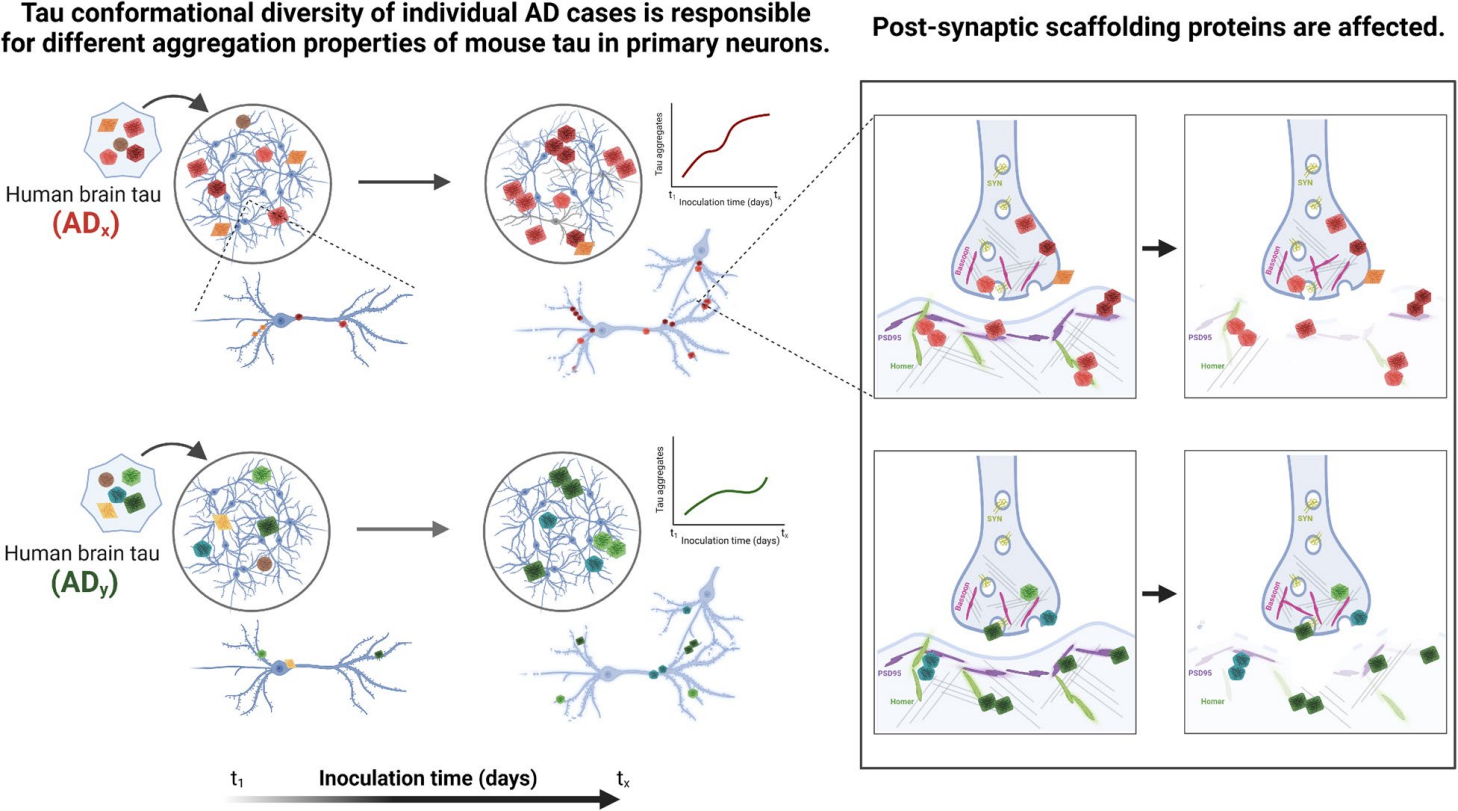
Schematic representation of data discussed in the presented research highlighting two main outcomes. In wild-type primary mouse neurons, aggregation of endogenous mouse tau is triggered by human AD-tau and more importantly, the aggregation properties of mouse tau (rate and conformation) are linked to the conformational diversity of the original individual AD cases used as inoculum. Thus, the propagation of misfolding and aggregation from human AD-tau to mouse tau occurs in a prion-like manner, and the diversity of human AD-tau species across individual AD cases can be transferred to primary neurons to model AD pathogenesis and its consequences at the level of cellular structures and mechanisms. The propagation of tau aggregation affects the synapses. The neurons with triggered tau pathology show reduced levels of synaptic connections, which may be linked to the disruption of the post-synaptic compartment and decreased levels of post-synaptic scaffolding proteins

Both experimental approaches provide direct evidence for the alterations in post-synaptic scaffolding protein levels, which indicate the earliest changes in somato-dendritic part of the synapses, and ultimately lead to the loss of synapses (Fig. 7).

## Discussion

Recent data showed that tau seeding potency to induce misfolding of tau monomers and to propagate the pathology is deeply implicated in heterogeneity across individual AD patients [17, 20–22]. We hypothesize that the heterogeneity of tau conformers in individual AD cases can be investigated by templated replication of mouse tau aggregation in primary neurons. To test the hypothesis, we applied mostly confocal microscopy and advanced biophysical techniques (CDI and CSA) to characterize the properties of newly formed aggregates of mouse tau triggered by Sarkosyl-insoluble tau from individual AD cases.

To extract human misfolded tau conformers from Sarkosyl-insoluble fractions of six individual AD frontal cortex tissue we applied PTA, which has a minimal effect on the conformational diversity of tau aggregates [35, 49]. These tau inocula varied greatly in levels of insoluble tau and their conformation (Table 3). Moreover, each AD-tau inoculum encompassed a spectrum of distinct conformers (CSA, Fig. 2A). These data are in agreement with the hypothesis of the diversity of misfolded tau species across individual AD cases and that the seeding-competent tau is likely to be a cloud of conformational entities such as oligomeric and soluble high-molecular weight forms, numerous transitional conformational states affected by post-translational modifications (PTMs), cofactors and physiochemical properties of aggregation environment [17, 18, 20, 28, 88, 89]. Even though a single set of two conformations of insoluble tau fibrils in AD have been identified by cryo-EM which are distinguished from tau fibrillar structures present in other tauopathies [23, 24], the presence of soluble forms of misfolded tau with high seeding activity can be monitored with another set of experimental approaches [17, 21, 26, 90, 91].

Primary neurons inoculated with each AD-tau sample formed detectable aggregates of endogenous mouse tau (Fig. 3). We used wild-type C57Bl/6Tac mice to obtain neuronal cultures for several critical reasons: (1) wt neurons have physiological levels of endogenous tau with no artefacts from overexpressed human tau, which can influence the rate of triggered mouse tau aggregation; (2) both mouse and human tau have the same amino acid sequence of tau repeat domain regions—the aggregation core; (3) mostly vary at the N-terminal part [71], which is an advantage in distinguishing mouse vs. human tau by applying antibodies specific against mouse and human tau, thus we can easily detect tau aggregates of mouse origin; (4) primary neurons have been and are still considered as the most valuable neuronal model due to relatively homogenous population of post-mitotic polarized neurons with distinguished somato-dendritic and axonal compartment and functional synaptic connectivity [92]; and (5) primary neurons also possess surface proteoglycans and LRP1 receptor facilitating neuronal internalization of extracellular tau [32, 93, 94], thus the physiological cellular machinery is encompassed in internalization of seeding-competent tau conformers imitating more native processes of tau transmission.

We had two main concerns when applying mouse primary neurons to investigate the heterogeneity of human tau aggregates. Firstly, human brains have a relatively equal ratio of 3R and 4R tau isoforms which serve as substrates for tau misfolding and aggregation during tau pathology progression in AD, which belongs to the 3R/4R tauopathy [73]. On the contrary, adult mice brains have mostly 4R tau isoforms [95]. Thus, we investigated the evolution of tau isoforms in primary mice neurons over time as more 3R tau is present in embryonic stages [66, 96]. The CDI data show that in the life span of neurons, upon inoculation at 7DIV, the neurons expressed both 3R and 4R tau in similar levels, but 3R tau isoform continued to decline thereafter to 20% at 14DIV and to 10% at 21DIV – the end-point of inoculation (Fig. 2C). Thus, we believe that the timeline for the treatment of primary neurons does not significantly affect distribution of 3R and 4R tau isoforms in tau aggregates formation [17]. The second concern of the use of primary neurons for this study was the cross-seeding barrier in tau aggregates transmission described in several research studies [97]. This phenomenon was described when neurons of wild-type origin and of transgenic mice with human P301L/S mutated tau were compared [30]. The higher rate of aggregation of mutated human tau variants can be partly explained by the faster incorporation of mutated tau, mostly due to its higher propensity for assembly into fibrils observed in vitro, formation of conformationally distinct aggregates, exclusively of 4R isoforms, and a different set of PTMs affecting the template seeding [37–40]. Recently, mouse models expressing both 3R and 4R murine tau [98] and mice expressing all six human tau isoforms against one a mouse tau knockout background [99] have been introduced, and we believe that this transgenic model will be an excellent tool in misfolded tau propagation studies.

To our best knowledge, this study provides the first evidence that the diversity of misfolded tau conformers of individual sporadic AD cases can be studied in wild-type mouse primary neurons as a cellular model. The application of FRET-biosensors [32] confirmed that tau in individual AD cases triggers distinct levels of aggregation of mutated tau constructs in HEK cells [21]. Mouse tau in wild-type primary neurons has been used as a substrate for templated prion-like propagation of tau misfolding when tau from various tauopathies, including AD, was used as a seed [66, 100]. Consistent with these studies, we were able to trigger the propagation of tau aggregation. Moreover, we monitored aggregation kinetics and conformational diversity of newly formed mouse tau aggregates after inoculation with all six AD-tau samples, which originally had various tau concentration levels and conformational properties. The degree of mouse tau aggregation was inoculum concentration-dependent and differs between inocula in the robustness of generating new aggregates and kinetics. The amount and structure of newly formed aggregates vary across the AD-tau samples applied. Our results show different outcomes than in [101], which can be related to original AD-tau heterogeneity. Interestingly, structures of the very ends of dendrites, MAP2-positive projections, were slightly altered, and dendritic debris occurred in tau aggregates to various extent for individual AD-tau cases. The aggregation of endogenous mouse tau is triggered only in the presence of human pathological tau since the control experiments with immunodepleted human AD-tau and monomeric mouse tau samples did not differ from untreated cultures.

Finally, we displayed the linear correlation of conformational data (D/N ratio of CDI) between original human AD-tau and newly generated mouse tau aggregates. Strikingly, we observed a statistically significant correlation between input human tau and output mouse tau conformers up to three days of inoculation. These data align with recently published data where 72 h seed kinetics monitored in FRET biosensors correlated with rate of AD progression [21]. We do not exclude the possibility that some tau aggregates, even more commonly shared among the AD-tau inocula, might be removed by neurons and only highly aggressive tau conformers that escape the proteasome apparatus of cells mainly contribute to templated mouse tau aggregation over time. Some samples displayed intra-sample variations, which we attribute to the non-uniformity of tau conformers within an individual sample as more to the presence of a cloud of tau aggregation with a range of conformational properties [17]. The correlation trend was not observed when neurons were incubated for 7 and 14 days (data not shown), which might indicate preferentially misfolding of more aggressive or lower energy tau conformers affected by binding molecular partners and biophysical properties of the aggregation environment inside mouse neurons.

In this study, we showed the template formation of misfolded tau aggregates can be triggered in wild-type mouse primary neurons and the properties like aggregation rates and conformations are dependent on the individual AD tau inoculum. Further, we investigated whether levels of synapses were affected by tau propagation in our neuronal system. It is known that the loss and disruption of synapses precede neuronal death and represent an early change in AD [6, 7, 102–104]. Moreover, both tau aggregation and synaptic dysfunction correlate with AD progression [12–14]. For proper functioning of synapses and effective chemical signaling, a proximity of both pre-synaptic and post-synaptic compartments is required. We applied the colocalization of Bassoon (pre-synaptic protein) and Homer (post-synaptic marker) and observed significantly reduced signal overlaps in all AD-tau treated neurons, which confirms loss of synapses. These results agree with numerous studies showing that tau pathology leads to synaptic loss [12–14]. Interestingly, we also noticed reduced levels of Homer (post-synaptic marker) and slightly elevated staining of Bassoon (pre-synaptic marker) as well as a disrupted actin staining pattern in human AD tau-treated neurons. These observations were confirmed with neuronal lysates where total levels of pre-synaptic markers Bassoon and synaptophysin were not significantly affected in contrast to the levels of post-synaptic markers PSD95 and Homer showing a decreasing trend. Our data imply that the post-synapse is disturbed to a higher extent than the pre-synaptic compartment. Although the precise mechanisms of pathogenic tau-mediated degeneration of synapses have still not been fully understood, some interesting data have been published in the last two decades. In mouse neurons transfected with monomeric human tau, postsynaptic markers including scaffolding protein PSD95 and presynaptic proteins of cytoskeletal matrix at active zone such as Bassoon were decreased [105]. Rat neurons bearing truncated human tau showed non-significant increase of Bassoon in presynaptic fractions [9]. In both studies, overall levels of synaptophysin were not affected but the number of pre-synaptically localized synaptophysin was reduced [9, 105]. Tau protein is mainly localized in axons but detected also in synapses [106, 107], and numerous physiological functions of tau are linked to the formation and function of the synapses [106, 108, 109]. In AD, propagation of pathological tau misfolding leads to tau missorting and synaptotoxicity [8, 9]. On the other hand, secreted tau seeds can be spread among synaptically connected neurons [103, 110] and Bassoon has been recently identified as a tau-seed interactor [111]. The disruption of functional and electrophysiological properties of synapses in neurons with tau pathology have been well documented in numerous studies [112–115]. In present study, the major primary objective was to apply new tools for investigating the evolution of mouse tau conformers in primary neurons inoculated with human AD tau and determine the time course of these effects, but we would like to investigate the molecular mechanism of differential synaptic effects and concurrent changes in electrophysiology in future projects. The relationship between spreading of tau species and synaptic dysfunction is likely to be a much more complex mechanism. In this study, we provide the first direct evidence that in non-overexpressing tau cell system, the significant loss of synapses is accompanied by reduction of postsynaptic scaffolding protein levels, when expression of pre-synaptic markers is not significantly affected.

## Conclusion

The heterogeneity of AD may complicate the success of available therapeutic interventions. The mechanisms behind clinical variability and different rates of AD progression have not been fully understood but is likely to be an intervention of multiple factors such as environment, genetics, and cellular mechanisms in individual patients. Tau pathology has become a hot topic in AD research as tau progression correlates with clinical progression and manifestation and leads to worsening of the symptoms. Lately, the diversity of seed-competent tau conformers was linked to various progression rates of AD. For the first time, we successfully applied wild-type mouse primary neurons to monitor diversity of tau conformers isolated from individual AD cases. Not only did we observe variations in individual AD-tau, but also the propagation of tau misfolding led to the loss of synapses due to reduced levels of postsynaptic terminals as an early event preceding neuronal death. We believe that our experiments conducted in physiological conditions of neuronal cultures help in understanding the diversity of tau conformers in AD and their link to synaptic loss.

### Supplementary Information

**Additional file 1: Figure S1.** Cytotoxicity effect of AD-tau inoculates and viability of the cells after treatment. (A) The viability of cells after 14 days inoculated with AD-tau and mouse tau (control) samples indicates minor decrease of ATP levels for inoculates AD3 and AD5-tau, and 0.2% Triton treated culture serves as a negative control for ATP assay. An exception is AD6-tau inoculum. When 45ng AD6-tau was applied, the ATP production was comparable to Triton-treated cells indicating cellular death (Multiple comparisons One-way ANOVA, medium vs. AD6-tau [45ng] and 0.2% Triton, p < 0.0001), thus we applied 15ng of AD6-tau instead of 45ng in all assays. One-way ANOVA with multiple comparisons for medium, 45ng of AD1-5 tau, and 15ng of AD6-tau showed some differences (medium vs. mouse Tau p = 0.932; vs. AD1 p = 0.786; vs. AD2 p = 0.615; vs. AD3 *p = 0.016; vs. AD4 p = 0.734; vs. AD5 *p = 0.017; vs. AD6 [15ng] p = 0.899), the levels of ATP after 14 days of post-inoculation decreased to 80% at the maximum compared to untreated samples (n = 5 per treatment, two independent experiments). (B) Cytotoxicity based on membrane disruption was measured as leakage of LDH into the medium by luminescence assay. (a) AD-tau and mouse tau (control) samples show minimal cytotoxicity effects after 14 days of inoculation. The levels of LDH in the medium were increased in cultures treated with AD1, AD2, and mouse tau maximum to one and a half levels of untreated group (medium). One-way ANOVA with multiple comparisons to medium (medium vs. mouse tau (Ctrl) ***p < 0.001; vs. ***AD1 p < 0.001; vs. AD2 *p = 0.0248; vs. AD3 p = 0.249; vs. AD4 p = 0.641; vs. AD5 p = 0.052; vs. AD6 p = 0.127; n = 5, two independent experiments combined). As a positive assay control served 0.2% Triton-treated cells. (b) The cultured wells corresponding to the inoculated wells display no cytotoxicity at 7DIV before treatment with AD-tau samples (One-way ANOVA, ns: p = 0.6593; n = 6 per corresponding treatment, two independent experiments combined). **Figure S2.** Presence of tau with pathological posttranslational modifications (truncation at D421 and phosphorylation at S202/T205) and human tau applied as an inoculum in hippocampal neurons treated with AD-tau for 14 days. (A) The truncated tau form known as TauC3 (truncation at D421) is present only in (a) 4% PFA fixed cells treated with AD-tau compared to medium. (b) Student t-test shows statistically significant increase of TauC3 tau in AD-tau treated cells compared to medium (p < 0.0001; n = 6 for each condition, 3 image frames from two independent experiments; particles counted from binary images created as threshold range 45-255 and the size of particles was set as 0.5 – infinity microns). (c) Ice-cold 100% methanol fixed cultures without soluble forms of tau lack the TauC3 positive insoluble conformers. Scale bars: 50 µm of large images, 10 µm of cropped areas. (B) Cultures with only insoluble tau (100% methanol fixed) were treated at 7DIV with AD-tau of human origin that is still detected at 21DIV (human tau = huTau, green) and many puncta positive of AT8 (red) shown that original human AD-tau and newly formed mouse tau are insoluble pathological tau conformers. Scale bars: 50 µm of large images, 30 µm of cropped areas. All images are presented as maximum intensities of 15 z-stacks (0.35µm each). **Figure S3.** Negative controls of mouse tau staining supporting the hypothesis that mouse tau aggregation occurs only in the presence of human AD-tau and additional validation of mouse- and human-specific tau antibodies (clones RTM47 and RTM49, respectively). (A) Cultures were treated at 7DIV with AD-tau inoculates (45ng of AD1-AD5 tau/well, 15ng of *AD6/well), inoculated for 14 days, and the fixation by ice-cold methanol followed. (a) In the left panel, the wells contained only medium and no cells, and the full staining protocol encompassing both primary and secondary antibodies against mouse tau (red) and MAP2 (cyan) showed no specific signal, thus the tau staining is mouse tau aggregate-specific and does not detect original human AD-tau inoculates. In the right panel, cells were treated according to the protocol, but no primary antibodies were applied during the staining procedure and no fluorescence signal for MAP2 and only tracer amount of non-specific signal in channel for secondary antibody conjugated with AF633 were detected in AD-tau treated samples, which we applied as a control for secondary antibodies nonspecific signal and background signal associated with impurities of AD-tau samples such as lipofuscin. We observed only nonspecific staining around the nuclei area in the red channel, which we also observed in the control untreated cells and is more associated with the detection in far red fluorescence channel. (b) The fully immunostained neurons inoculated with 45ng of AD5 from Fig 3 is inserted for evaluation of positive fluorescent signal in both mouse tau and MAP2 channels. (c) The threshold binary images of fluorescent channel applied with mouse tau in inoculated fully stained neurons from Fig 3 (red outline, first column), inoculated wells with no seeded wells (second column), and inoculated neurons immunostained in the absence of primary antibodies (third column) both from A-a show signal specific to aggregated mouse tau. (B) Mouse-specific (clone: RTM47) and human-specific (clone; RTM49) tau antibodies (both diluted 1:4000) were evaluated by western blots loaded with human recombinant 3R and 4R tau (40ng/line, rPeptide), control human and mouse brain homogenates (10µg/line), and cell lysate of 21DIV mice cortical neurons (5µg/line), chemiluminescence detection under same conditions for both membranes. (C) Both antibodies were evaluated also with immunostaining of untreated 21DIV cultures of mice cortical neurons using standard fixation protocol (4% paraformaldehyde) enabling us detecting axonally localized tau. Mice neurons are stained only with mouse-specific and not with human-specific tau antibody. (D) The mouse tau (control) sample as an inoculum for mouse tau aggregation in neurons by confocal microscopy was evaluated by staining and confocal microscopy. When compared with untreated neurons, we saw minimal fluorescence signal of aggregated mouse tau (red) and no alterations in MAP2 staining pattern (cyan). All images are presented as maximum intensities of 35 z-stacks (0.35µm each), scale bars: 50 µm of large images, 20 µm of cropped areas. The staining for aggregated tau was performed after ice-cold methanol fixation. **Figure S4.** Detection of the increased rate of tau aggregation is related to endogenous mouse tau aggregation triggered by AD-tau inoculates. (Aa,b) Mouse tau aggregation was significantly reduced in the immunodepleted-AD5 (ID-AD5) sample compared with the original AD5-tau sample. No aggregation was detected in western blot 1h after incubation, but only AD5-tau triggered mouse tau aggregation was observed within 14d of post-inoculation compared to all controls, ID-AD5- and mouse tau (control)-inoculated neurons, and untreated cultures (medium). The western blots are from the same membrane. (B) The expanded graph of Fig5 (D, c): the density of HMW mouse tau (> 50kDa) normalized to PonceauS staining in sarkosyl-insoluble pellets from cell lysis was calculated from western blots with specific-mouse tau antibody in all time points (1h, 3d, 7d, and 14d of inoculation, n = 3 for each inoculum and time-point). The trend of tau aggregation curves for cultures inoculated with individual AD-tau samples corresponds to aggregation rate patterns measured in CDI (from Fig5-Ba). **Figure S5.** Pre- and post-synaptic markers in control cultures (ID-AD5 and mouse tau inoculated) over time post-inoculation measured and calculated from western blots. The linear regression of signals expressed as densities of target protein bands normalized to GAPDH and then as N-fold change to medium-treated cells and combined from three independent experiments. There is no statistically significant difference in both pre-and post-synaptic markers levels at different time points for control-treated cultures (Synaptophysin: p = 0.765; Bassoon: p = 0.203; PSD95: p = 0.283; Homer: p = 0.678), n = 6 per time point, three of Mo Tau treated, three of ID-AD5 treated cultures).

## Data Availability

All data associated with this study are present in the paper or the Additional file 1.
