# Supplementary material for: Evolving prion-like tau conformers differentially alter postsynaptic proteins in neurons inoculated with distinct isolates of Alzheimer’s disease tau
Source: Cell Biosci. 2023 Sep 18;13:174. doi: 10.1186/s13578-023-01133-0 (PMC10507869; doi:10.1186/s13578-023-01133-0)
Supplement: Supplementary file 1 — Additional file 1: Figure S1. Cytotoxicity effect of AD-tau inoculates and viability of the cells after treatment. (A) The viability of cells after 14 days inoculated with AD-tau and mouse tau (control) samples indicates minor decrease of ATP levels for inoculates AD3 and AD5-tau, and 0.2% Triton treated culture serves as a negative control for ATP assay. An exception is AD6-tau inoculum. When 45ng AD6-tau was applied, the ATP production was comparable to Triton-treated cells indicating cellular death (Multiple comparisons One-way ANOVA, medium vs. AD6-tau [45ng] and 0.2% Triton, p < 0.0001), thus we applied 15ng of AD6-tau instead of 45ng in all assays. One-way ANOVA with multiple comparisons for medium, 45ng of AD1-5 tau, and 15ng of AD6-tau showed some differences (medium vs. mouse Tau p = 0.932; vs. AD1 p = 0.786; vs. AD2 p = 0.615; vs. AD3 *p = 0.016; vs. AD4 p = 0.734; vs. AD5 *p = 0.017; vs. AD6 [15ng] p = 0.899), the levels of ATP after 14 days of post-inoculation decreased to 80% at the maximum compared to untreated samples (n = 5 per treatment, two independent experiments). (B) Cytotoxicity based on membrane disruption was measured as leakage of LDH into the medium by luminescence assay. (a) AD-tau and mouse tau (control) samples show minimal cytotoxicity effects after 14 days of inoculation. The levels of LDH in the medium were increased in cultures treated with AD1, AD2, and mouse tau maximum to one and a half levels of untreated group (medium). One-way ANOVA with multiple comparisons to medium (medium vs. mouse tau (Ctrl) ***p < 0.001; vs. ***AD1 p < 0.001; vs. AD2 *p = 0.0248; vs. AD3 p = 0.249; vs. AD4 p = 0.641; vs. AD5 p = 0.052; vs. AD6 p = 0.127; n = 5, two independent experiments combined). As a positive assay control served 0.2% Triton-treated cells. (b) The cultured wells corresponding to the inoculated wells display no cytotoxicity at 7DIV before treatment with AD-tau samples (One-way ANOVA, ns: p = 0.6593; n = 6 per corresponding treatment, t [file 13578_2023_1133_MOESM1_ESM.docx]

**Supplementary material:**

**Evolving prion-like tau conformers differentially alter postsynaptic proteins in neurons inoculated with distinct isolates of Alzheimer’s disease tau.**

**Lenka Hromadkova^1^, Chae Kim^1^, Tracy Haldiman^1^, Lihua Peng^1^, Xiongwei Zhu^1,2^, Mark Cohen^1,4^, Rohan de Silva^5^, Jiri G. Safar^1,2,3^**

**Affiliations**

Departments of ^1^Pathology, ^2^Neurology, ^3^Neuroscience, and ^4^National Prion Disease Pathology Surveillance Center, Case Western Reserve University School of Medicine, Cleveland, OH 44106, USA;

^5^Reta Lila Weston Institute, UCL Queen Square Institute of Neurology, London, WC1N 1PJ, UK;

*Address correspondence to: Jiri G. Safar, Department of Pathology, Case Western Reserve University, 2085 Adelbert Rd, Cleveland, OH 44106. Tel: (216) 368-4609; Fax: (216) 368-4090; E-mail: [jiri.safar@case.edu](mailto:jiri.safar@case.edu)

Supplement Figure 1:


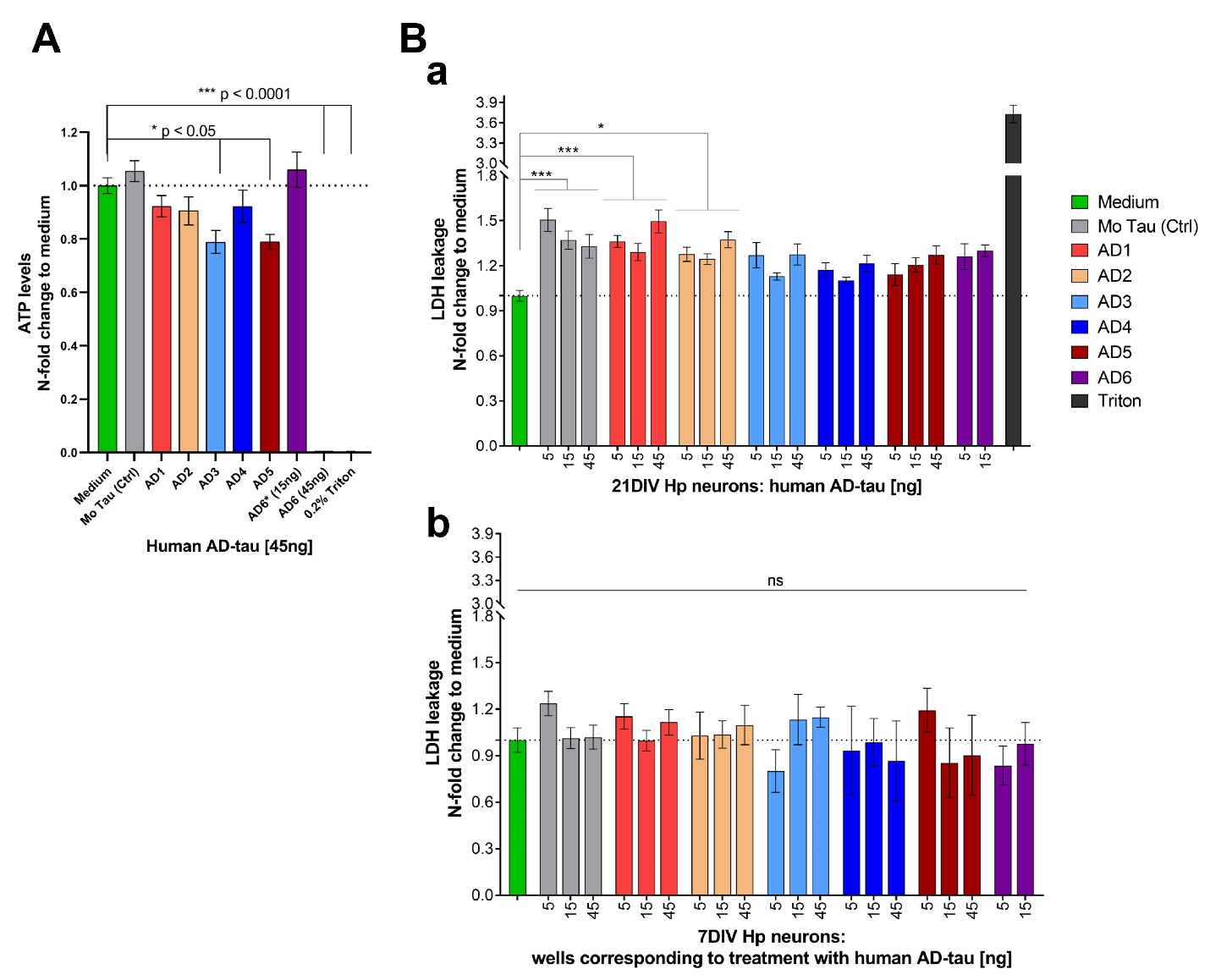


**Supplement Figure 1:** Cytotoxicity effect of AD-tau inoculates and viability of the cells after treatment. **(A)** The viability of cells after 14 days inoculated with AD-tau and mouse tau (control) samples indicates minor decrease of ATP levels for inoculates AD3 and AD5-tau, and 0.2% Triton treated culture serves as a negative control for ATP assay. An exception is AD6-tau inoculum. When 45ng AD6-tau was applied, the ATP production was comparable to Triton-treated cells indicating cellular death (Multiple comparisons One-way ANOVA, medium vs. AD6-tau [45ng] and 0.2% Triton, p < 0.0001), thus we applied 15ng of AD6-tau instead of 45ng in all assays. One-way ANOVA with multiple comparisons for medium, 45ng of AD1-5 tau, and 15ng of AD6-tau showed some differences (medium vs. mouse Tau p = 0.932; vs. AD1 p = 0.786; vs. AD2 p = 0.615; vs. AD3 *p = 0.016; vs. AD4 p = 0.734; vs. AD5 *p = 0.017; vs. AD6 [15ng] p = 0.899), the levels of ATP after 14 days of post-inoculation decreased to 80% at the maximum compared to untreated samples (n = 5 per treatment, two independent experiments). **(B)** Cytotoxicity based on membrane disruption was measured as leakage of LDH into the medium by luminescence assay. **(a)** AD-tau and mouse tau (control) samples show minimal cytotoxicity effects after 14 days of inoculation. The levels of LDH in the medium were increased in cultures treated with AD1, AD2, and mouse tau maximum to one and a half levels of untreated group (medium). One-way ANOVA with multiple comparisons to medium (medium vs. mouse tau (Ctrl) ***p < 0.001; vs. ***AD1 p < 0.001; vs. AD2 *p = 0.0248; vs. AD3 p = 0.249; vs. AD4 p = 0.641; vs. AD5 p = 0.052; vs. AD6 p = 0.127; n = 5, two independent experiments combined). As a positive assay control served 0.2% Triton-treated cells. **(b)** The cultured wells corresponding to the inoculated wells display no cytotoxicity at 7DIV before treatment with AD-tau samples (One-way ANOVA, ns: p = 0.6593; n = 6 per corresponding treatment, two independent experiments combined).

Supplement Figure 2:


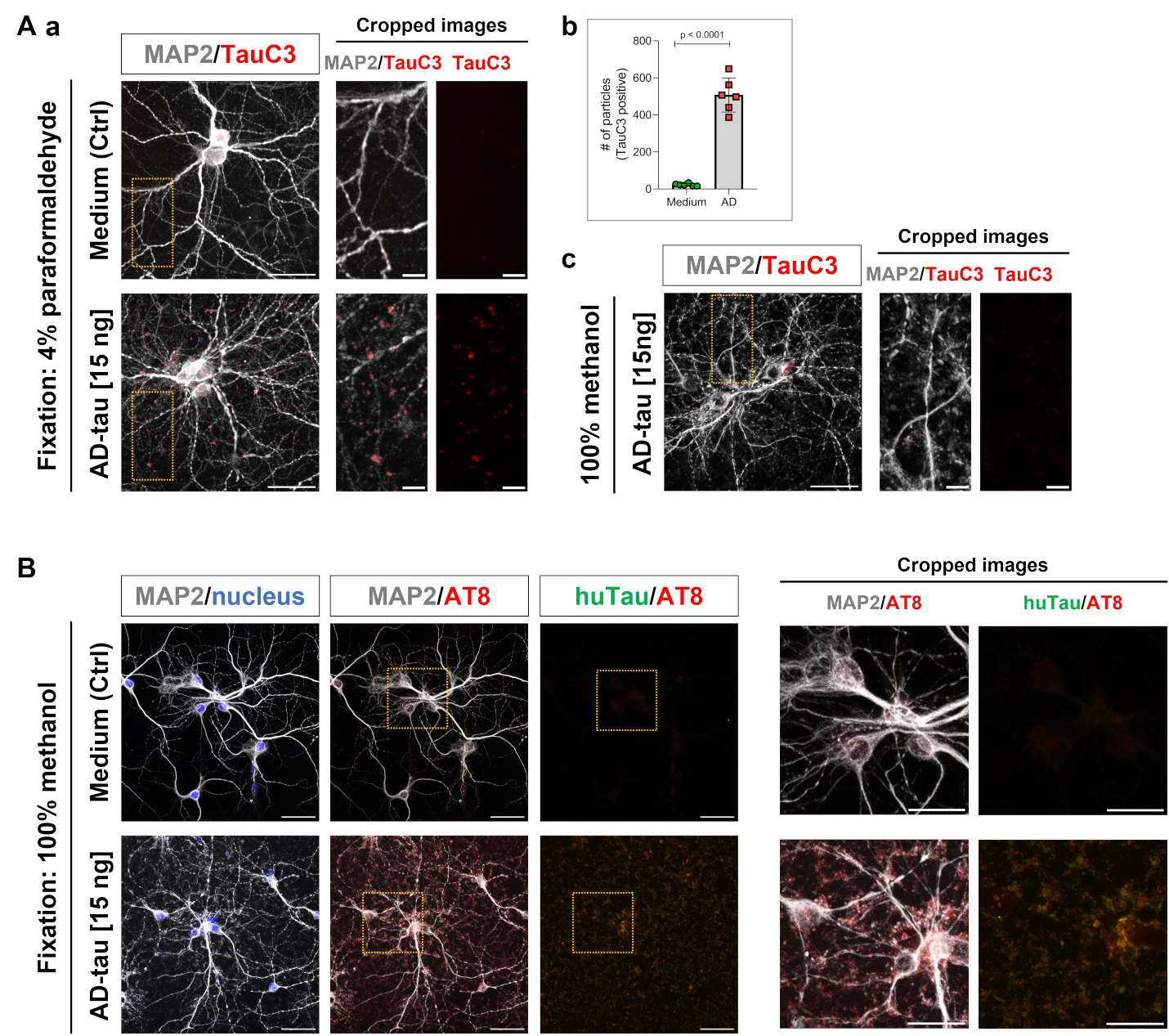


**Supplement Figure 2:** Presence of tau with pathological posttranslational modifications (truncation at D421 and phosphorylation at S202/T205) and human tau applied as an inoculum in hippocampal neurons treated with AD-tau for 14 days. **(A)** The truncated tau form known as TauC3 (truncation at D421) is present only in **(a)** 4% PFA fixed cells treated with AD-tau compared to medium. **(b)** Student t-test shows statistically significant increase of TauC3 tau in AD-tau treated cells compared to medium (p < 0.0001; n = 6 for each condition, 3 image frames from two independent experiments; particles counted from binary images created as threshold range 45-255 and the size of particles was set as 0.5 – infinity microns). **(c)** Ice-cold 100% methanol fixed cultures without soluble forms of tau lack the TauC3 positive insoluble conformers. Scale bars: 50 µm of large images, 10 µm of cropped areas. **(B)** Cultures with only insoluble tau (100% methanol fixed) were treated at 7DIV with AD-tau of human origin that is still detected at 21DIV (human tau = huTau, green) and many puncta positive of AT8 (red) shown that original human AD-tau and newly formed mouse tau are insoluble pathological tau conformers. Scale bars: 50 µm of large images, 30 µm of cropped areas. All images are presented as maximum intensities of 15 z-stacks (0.35µm each).

Supplement Figure 3:


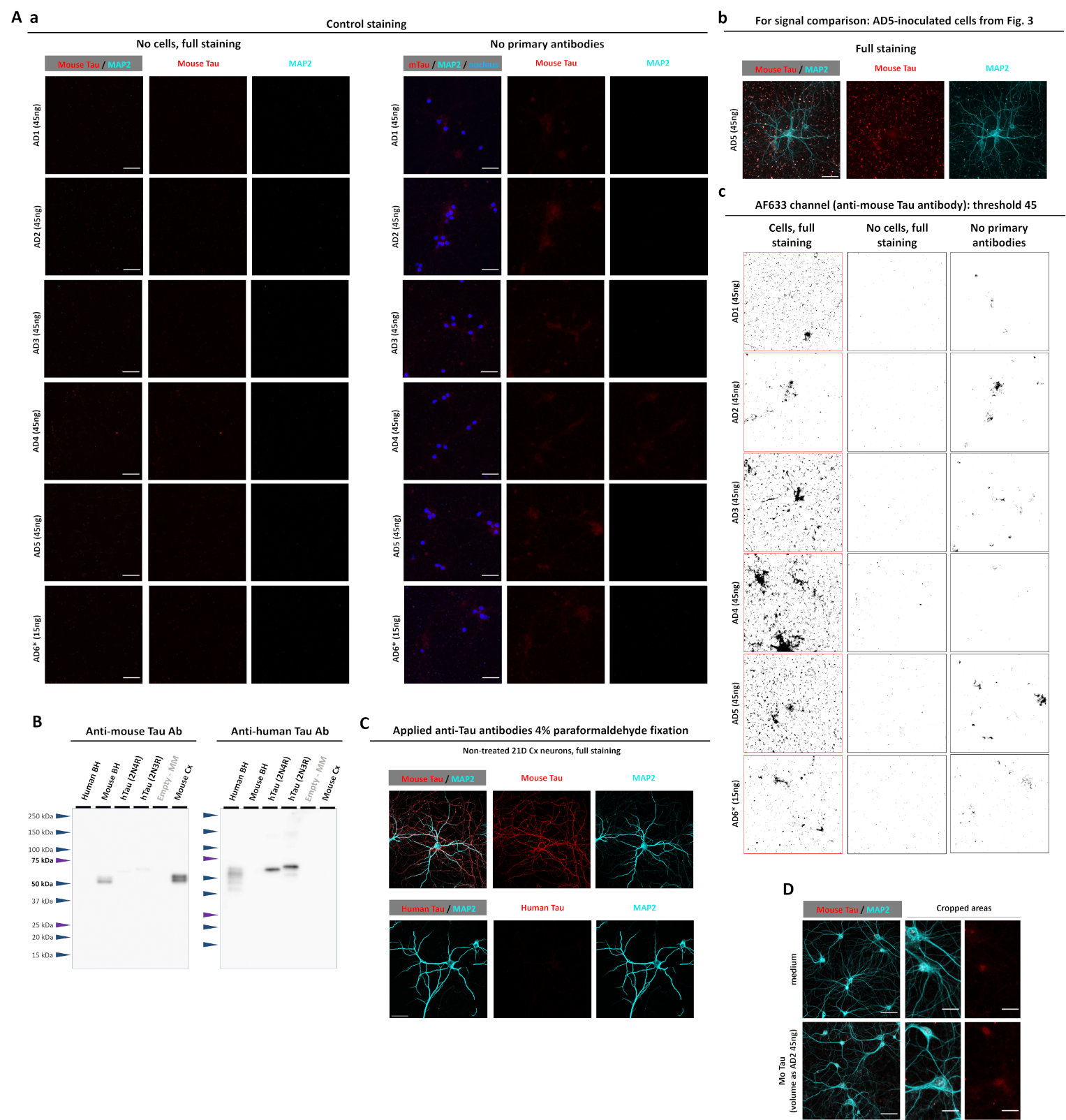


**Supplement Figure 3:** Negative controls of mouse tau staining supporting the hypothesis that mouse tau aggregation occurs only in the presence of human AD-tau and additional validation of mouse- and human-specific tau antibodies (clones RTM47 and RTM49, respectively). **(A)** Cultures were treated at 7DIV with AD-tau inoculates (45ng of AD1-AD5 tau/well, 15ng of *AD6/well), inoculated for 14 days, and the fixation by ice-cold methanol followed. **(a)** In the left panel, the wells contained only medium and no cells, and the full staining protocol encompassing both primary and secondary antibodies against mouse tau (red) and MAP2 (cyan) showed no specific signal, thus the tau staining is mouse tau aggregate-specific and does not detect original human AD-tau inoculates. In the right panel, cells were treated according to the protocol, but no primary antibodies were applied during the staining procedure and no fluorescence signal for MAP2 and only tracer amount of non-specific signal in channel for secondary antibody conjugated with AF633 were detected in AD-tau treated samples, which we applied as a control for secondary antibodies nonspecific signal and background signal associated with impurities of AD-tau samples such as lipofuscin. We observed only nonspecific staining around the nuclei area in the red channel, which we also observed in the control untreated cells and is more associated with the detection in far red fluorescence channel. **(b)** The fully immunostained neurons inoculated with 45ng of AD5 from Fig 3 is inserted for evaluation of positive fluorescent signal in both mouse tau and MAP2 channels. **(c)** The threshold binary images of fluorescent channel applied with mouse tau in inoculated fully stained neurons from Fig 3 (red outline, first column), inoculated wells with no seeded wells (second column), and inoculated neurons immunostained in the absence of primary antibodies (third column) both from A-a show signal specific to aggregated mouse tau. **(B)** Mouse-specific (clone: RTM47) and human-specific (clone; RTM49) tau antibodies (both diluted 1:4000) were evaluated by western blots loaded with human recombinant 3R and 4R tau (40ng/line, rPeptide), control human and mouse brain homogenates (10µg/line), and cell lysate of 21DIV mice cortical neurons (5µg/line), chemiluminescence detection under same conditions for both membranes. **(C)** Both antibodies were evaluated also with immunostaining of untreated 21DIV cultures of mice cortical neurons using standard fixation protocol (4% paraformaldehyde) enabling us detecting axonally localized tau. Mice neurons are stained only with mouse-specific and not with human-specific tau antibody. **(D)** The mouse tau (control) sample as an inoculum for mouse tau aggregation in neurons by confocal microscopy was evaluated by staining and confocal microscopy. When compared with untreated neurons, we saw minimal fluorescence signal of aggregated mouse tau (red) and no alterations in MAP2 staining pattern (cyan). All images are presented as maximum intensities of 35 z-stacks (0.35µm each), scale bars: 50 µm of large images, 20 µm of cropped areas. The staining for aggregated tau was performed after ice-cold methanol fixation.

Supplement Figure 4:


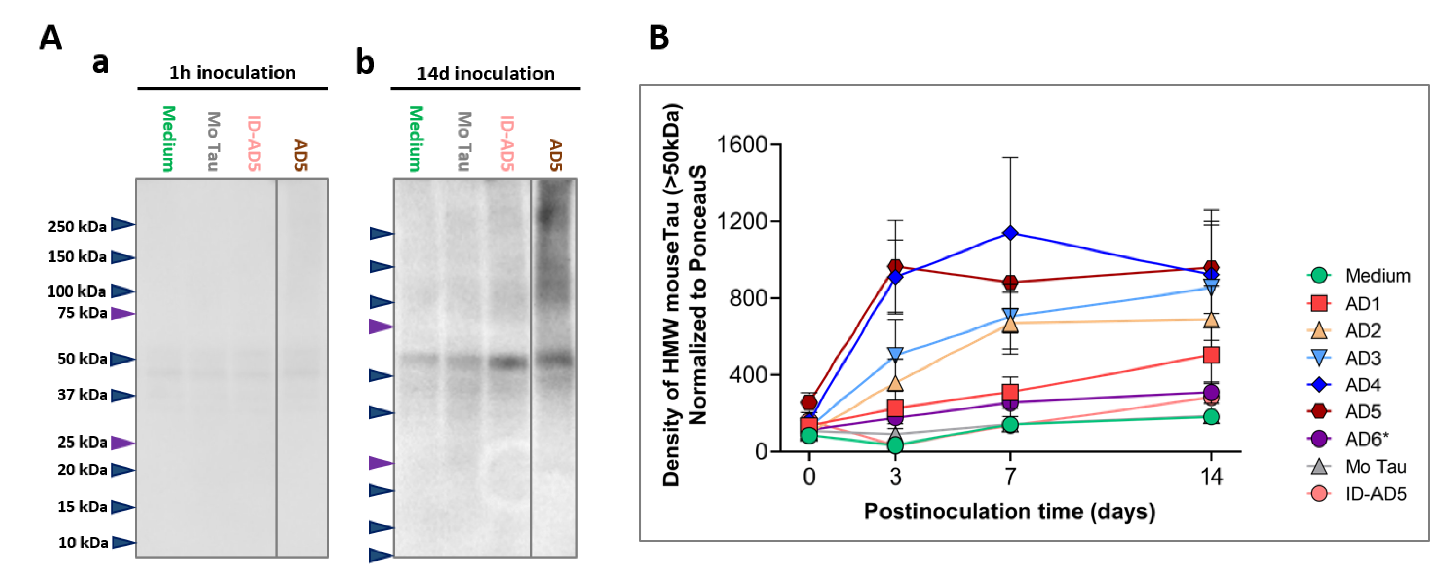


**Supplement Figure 4:** Detection of the increased rate of tau aggregation is related to endogenous mouse tau aggregation triggered by AD-tau inoculates. **(Aa,b)** Mouse tau aggregation was significantly reduced in the immunodepleted-AD5 (ID-AD5) sample compared with the original AD5-tau sample. No aggregation was detected in western blot 1h after incubation, but only AD5-tau triggered mouse tau aggregation was observed within 14d of post-inoculation compared to all controls, ID-AD5- and mouse tau (control)-inoculated neurons, and untreated cultures (medium). The western blots are from the same membrane. **(B)** The expanded graph of Fig5 (D, c): the density of HMW mouse tau (> 50kDa) normalized to PonceauS staining in sarkosyl-insoluble pellets from cell lysis was calculated from western blots with specific-mouse tau antibody in all time points (1h, 3d, 7d, and 14d of inoculation, n = 3 for each inoculum and time-point). The trend of tau aggregation curves for cultures inoculated with individual AD-tau samples corresponds to aggregation rate patterns measured in CDI (from Fig5-Ba).

Supplement Figure 5:


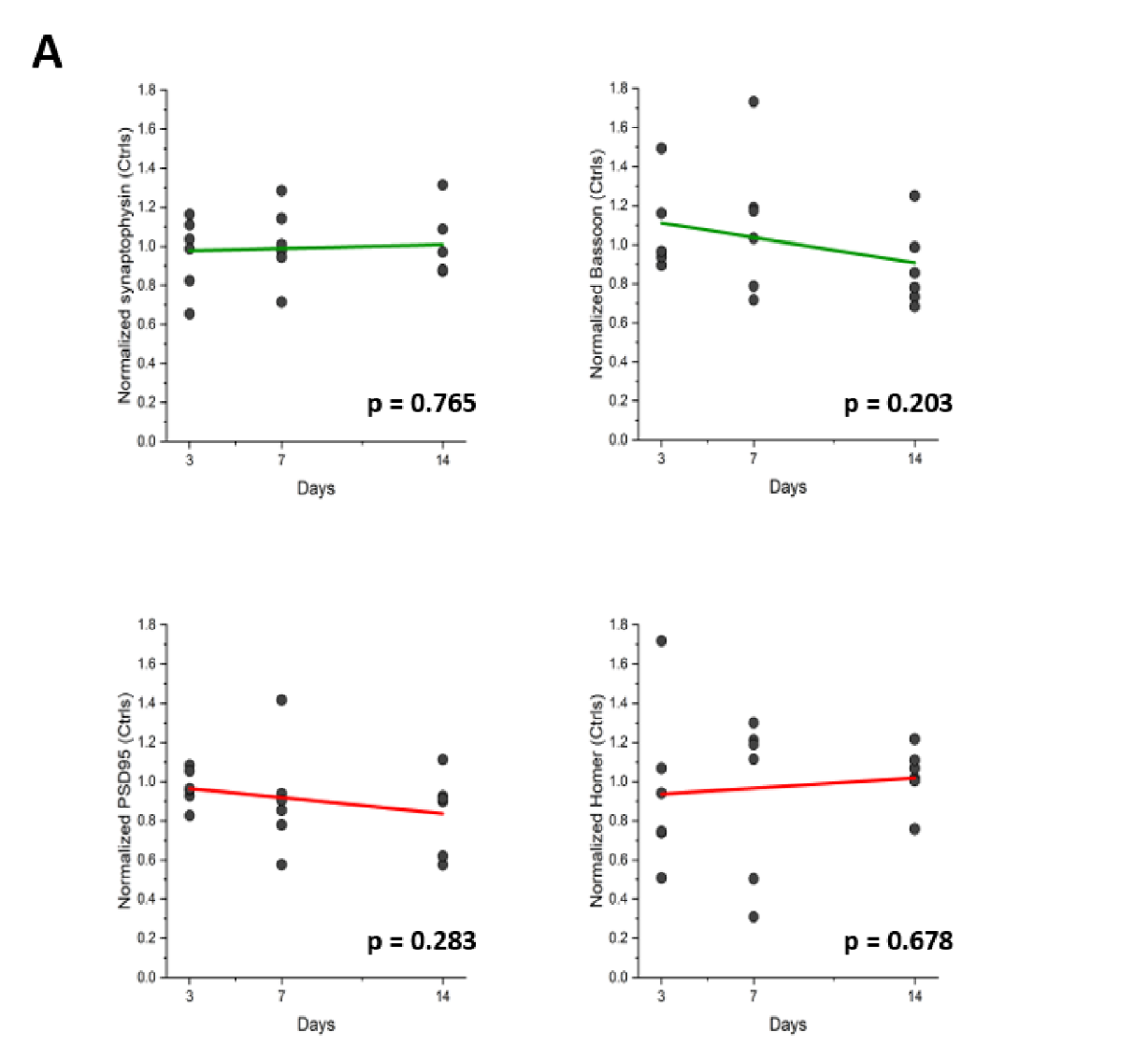


**Supplement Figure 5:** Pre- and post-synaptic markers in control cultures (ID-AD5 and mouse tau inoculated) over time post-inoculation measured and calculated from western blots. The linear regression of signals expressed as densities of target protein bands normalized to GAPDH and then as N-fold change to medium-treated cells and combined from three independent experiments. There is no statistically significant difference in both pre-and post-synaptic markers levels at different time points for control-treated cultures (Synaptophysin: p = 0.765; Bassoon: p = 0.203; PSD95: p = 0.283; Homer: p = 0.678), n = 6 per time point, three of Mo Tau treated, three of ID-AD5 treated cultures).
